# Contemporary limnology of the rapidly changing glacierized watershed of the world’s largest High Arctic lake

**DOI:** 10.1038/s41598-019-39918-4

**Published:** 2019-03-14

**Authors:** K. A. St. Pierre, V. L. St. Louis, I. Lehnherr, S. L. Schiff, D. C. G. Muir, A. J. Poulain, J. P. Smol, C. Talbot, M. Ma, D. L. Findlay, W. J. Findlay, S. E. Arnott, Alex S. Gardner

**Affiliations:** 1 Department of Biological Sciences, University of Alberta, Edmonton, AB T6G 2E9 Canada; 2 Department of Geography, University of Toronto at Mississauga, Mississauga, ON L5L 1C6 Canada; 3 Department of Earth and Environmental Science, University of Waterloo, Waterloo, ON N2L 3G1 Canada; 4 Canada Centre for Inland Waters, Environment and Climate Change Canada, Burlington, ON L7S 1A1 Canada; 5 Department of Biology, University of Ottawa, Ottawa, ON K1N 6N5 Canada; 6 Department of Biology, Queen’s University, Kingston, ON K7L 3N6 Canada; 7 Plankton R Us, Winnipeg, MB R2N 1M1 Canada; 8 NASA Jet Propulsion Laboratory, California Institute of Technology, Pasadena, California 91109 USA

## Abstract

Glacial runoff is predicted to increase in many parts of the Arctic with climate change, yet little is known about the biogeochemical impacts of meltwaters on downstream freshwater ecosystems. Here we document the contemporary limnology of the rapidly changing glacierized watershed of the world’s largest High Arctic lake (Lake Hazen), where warming since 2007 has increased delivery of glacial meltwaters to the lake by up to 10-times. Annually, glacial meltwaters accounted for 62–98% of dissolved nutrient inputs to the lake, depending on the chemical species and year. Lake Hazen was a strong sink for NO_3_^−^-NO_2_^−^, NH_4_^+^ and DOC, but a source of DIC to its outflow the Ruggles River. Most nutrients entering Lake Hazen were, however, particle-bound and directly transported well below the photic zone via dense turbidity currents, thus reinforcing ultraoligotrophy in the lake rather than overcoming it. For the first time, we apply the land-to-ocean aquatic continuum framework in a large glacierized Arctic watershed, and provide a detailed and holistic description of the physical, chemical and biological limnology of the rapidly changing Lake Hazen watershed. Our findings highlight the sensitivity of freshwater ecosystems to the changing cryosphere, with implications for future water quality and productivity at high latitudes.

## Introduction

Global glacier (non-ice sheet) volume is expected to decline by 29–41% by 2100^1^, with coincident changes to downstream runoff^2,3^. This loss is particularly important in the Arctic, where ~58% of the world’s glaciers are located^4^ and climate change is predicted to be most intense^5^. Glaciers are important archives of nutrients^6^ and contaminants^7–9^ that were transported and deposited to high latitude regions over centuries or millennia. Thus, as glaciers melt, they become potentially important sources of these compounds to downstream ecosystems^10–12^. Further, as meltwaters travel across poorly consolidated proglacial zones, they can entrain large quantities of fine, chemically reactive sediments^12,13^. Whereas the direct impacts of glacial melt on the biogeochemistry of nearshore marine environments has been highlighted^14–16^, we understand little of the hydrological-biogeochemical coupling across changing Arctic landscapes, which hinders our ability to predict the consequences of climate change on valuable northern freshwater function, resources and services. In more temperate latitudes, for example, it is well established that freshwater quality changes dramatically in response to biological (e.g., primary production, respiration), physical (e.g., sediment burial) and chemical processes (e.g., oxidation, reduction) as water flows across landscapes on its way to the ocean^17^.

The land to ocean aquatic continuum (LOAC) framework acknowledges these inherent connections between terrestrial, freshwater and marine systems^18^. The LOAC framework has hitherto only been applied in temperate forested and agricultural watersheds (e.g., refs^17,19^,) and has focused primarily on rivers and streams, even though lakes are integral parts of the continuum, acting to retain and process organic matter, carbon and nutrients before waters flow further downstream^17,20,21^. In glacierized watersheds, lakes are becoming increasingly dynamic components of the LOAC as their area and numbers increase globally in response to rapid glacial melt^22,23^. These proglacial lakes are typically located in sparsely vegetated catchments with poorly developed soils, which limits allochthonous carbon inputs but facilitates the entrainment of easily mobilized mineral sediments and nutrients.

**Figure 1:**
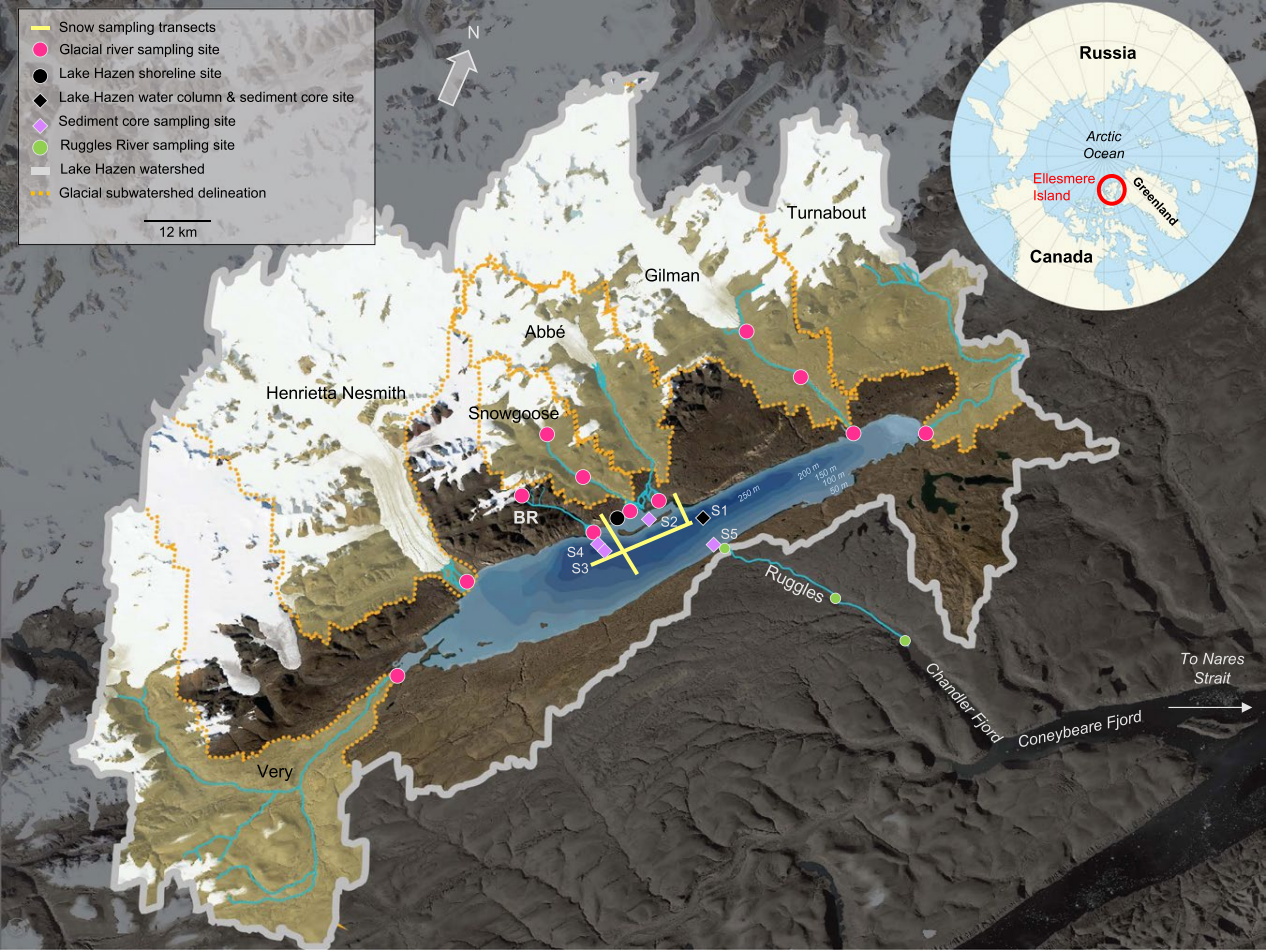
Map of the Lake Hazen watershed exemplifying the glacierized land-to-ocean aquatic continuum. The solid grey and orange-dotted lines delineate the entire Lake Hazen watershed and the glacial sub-watersheds, respectively. Sampling sites are highlighted by type. Sediment coring sites (S1–5) are described in Table S8. BR, Blister River. Background image contains modified Copernicus Sentinel-2 data [2016] processed by Sentinel Hub; watershed delineations generated in ArcGIS 10.5 using the hydrology tools. Background of inset map from Wikimedia Commons (https://commons.wikimedia.org/wiki/File:Arctic_Ocean_location_map.svg; CC BY-SA 3.0 - https://creativecommons.org/licenses/by-sa/3.0).

Here we use the Lake Hazen watershed (81–82°N) as a model system to examine a High Arctic LOAC, from glacial headwaters through proglacial river valleys, into a large lake that then drains out to the coast (Fig. 1, Table S1). The Lake Hazen watershed has recently undergone significant changes in response to a 1 °C increase in air temperature, including a 10-fold increase in glacier runoff with concomitant increases in sedimentation, as well as a reduction in the ice-covered season on the lake^10^. The limnological function of the lake has, however, never been described in detail. Our objectives were to: (a) describe the physical, chemical and biological limnology of the Lake Hazen watershed within the context of a glacierized LOAC; (b) quantify the impact of in-lake processing of glacial meltwaters using hydrological input-output budgets; (c) describe water column plankton community assemblages and sediment biogeochemical processes in relation to glacial inputs; and, (d) apply ecosystem stoichiometry to identify locations of nutrient mobilization and retention across the glacierized LOAC. This study focuses primarily on the biogeochemical processing of carbon and macronutrients within the system (nitrogen, N; phosphorus, P), and micronutrients iron (as total iron; TFe) and dissolved silica (dSiO_2_) are also discussed. Sulfate (SO_4_^2−^) is presented as a conservative tracer throughout.

## Results and Discussion

### Watershed hydrology

Total hydrological inputs to the lake were 1.063 ± 0.027 km^3^ in 2015 and 0.400 ± 0.097 km^3^ in 2016. Mean annual snowmelt inputs were 0.079 ± 0.013 km^3^ over the lake area, assuming no sublimation (Table 1), while landscape (non-glacierized, non-lake area) snowmelt contributions to Lake Hazen were 0.075 ± 0.025 km^3^ (means of 2013, 2015, 2017). The dominant annual input was thus glacial runoff, accounting for approximately 86.7% and 67.1% of annual hydrological inputs in 2015 and 2016, respectively. Total glacial runoff was 3.37-times greater in 2015 (0.979 km^3^) than in 2016 (0.291 km^3^). Three glacial rivers (Henrietta Nesmith, Gilman and Very) cumulatively accounted for ~70% of glacial runoff into Lake Hazen. With cold-based margins, most of the meltwaters from the Northern Ellesmere Icefield flow along the top of the glaciers and ice marginal channels before being discharged to the proglacial zone, thus limiting contact with glacier beds. Complete ice-off on Lake Hazen occurred around 4-Aug-2015 and 8-Aug-2016 in the two summer study years.

**Table 1:** Hydrological nutrient mass balances (loads in metric tons yr^−1^ ± SE) for Lake Hazen in 2015 and 2016.

	Year	Hydrological inputs				Outputs	
		Snow on lake^a^	Snow from land^b^	Glacial rivers	Total inputs	Ruggles River	% diff^c^.
Water (km^3^)	2015	0.069 ± 0.015	0.055 ± 0.012	0.948	1.07 ± 0.027	1.07 ± 0.027	−
	2016	0.060 ± 0.008	0.059 ± 0.089	0.281	0.400 ± 0.097	0.400 ± 0.097	−
**NH**_**4**_^+^	**2015**	**0.584** ± **0.115**	**1.67** ± **0.454**	**22.9** ± **15.9**	**25.2** ± **16.5**	**3.79** ± **1.38**	−71.1*
	**2016**	**0.551** ± **0.092**	**1.48** ± **0.371**	**5.42** ± **2.15**	**7.45** ± **2.61**	**1.13** ± **0.411**	−63.6*
**NO**_**3**_^−^-**NO**_**2**_^−^	**2015**	**2.81** ± **0.611**	**5.63** ± **1.89**	**35.1** ± **4.84**	**43.6** ± **7.34**	**12.0** ± **7.59**	−33.7*
	**2016**	**2.22** ± **0.361**	**4.98** ± **1.59**	**12.0** ± **1.34**	**19.2** ± **3.29**	**3.57** ± **2.26**	−40.4*
SO_4_^2−^	2015	73.0 ± 0.016	993 ± 235	13400 ± 2420	14420 ± 2661	9400 ± 1440	−15.5
	2016	77.8 ± 0.016	879 ± 188	3580 ± 411	4540 ± 599	2790 ± 428	−6.66
TDP^d^	2015	0.177 ± 0.037	1.00 ± 0.309	—	—	—	—
	2016	0.134 ± 0.029	1.08 ± 0.282	—	—	—	—
dSiO_2_	2015	5.88 ± 0.001	16.0 ± 3.21	181 ± 59.2	203 ± 62.4	246 ± 75.8	+15.4
	2016	5.09 ± 0.001	14.2 ± 2.44	56.9 ± 11.0	76.2 ± 13.5	73.2 ± 22.5	+15.5
**DIC**	**2015**	**139** ± **0.028**	**102** ± **21.0**	**6310** ± **474**	**6550** ± **495**	**9700** ± **2470**	+24.9*
	**2016**	**117** ± **0.019**	**88.0** ± **15.4**	**1920** ± **117**	**2120** ± **132**	**2880** ± **735**	+31.7*
**DOC**	**2015**	**6.92** ± **0.002**	**0.087** ± **0.023**	**364** ± **59.6**	**371** ± **59.6**	**203** ± **55.9**	−23.7*
	2016	13.3 ± 0.003	0.077 ± 0.018	109 ± 14.5	123 ± 14.6	60.6 ± 16.6	−17.6

^a^Snow chemistry from 2015 only applied to 2015, and mean snowpack AWV across 2013, 2015, 2017 applied to 2016.

^b^Snowmelt volume calculated assuming 16% runoff of snowpack on landscape (see methods).

^c^Difference between total mean hydrological inputs and outputs by the Ruggles River. Budgets for which output load ranges are outside of the range of inputs (bolded and *), suggesting an important annual source (% difference >0) or sink (% difference <0) within the lake.

^d^TDP was only above detection in snow, and as such glacial river and Ruggles River compartment fluxes could not be calculated.

### Nutrient concentrations in major hydrological inputs to Lake Hazen

#### Snowmelt on the lake and from the landscape

Snow was circumneutral (pH = 7.47 ± 0.126) and contained some particles (TSS = 106 ± 14.9 mg/L; Table S2). Particulates in Arctic snowpacks can originate locally from aeolian processes acting on exposed soils on the desert landscape and from distant sources via long-range atmospheric transport^24^. Concentrations of DIN in snowpacks were 50.4 ± 3.12 µg/L, most of which was NO_3_^−^-NO_2_^−^. TP and TDP concentrations in snow were 45 ± 4 µg/L and 2.52 ± 0.308 µg/L, respectively. Carbon concentrations in the snowpack were generally low and dominated by PC (2.10 ± 0.194 mg-C/L).

Snowmelt streams were cold (2.76 ± 1.71 °C), and circumneutral (pH = 7.20 ± 0.18), with low suspended sediment concentrations (54.9 ± 23.8 mg/L). Analyte concentrations were generally higher than in snowpacks, reflecting a combination of snowpack sublimation and chemical elution^25^. DIN concentrations were highest in snowmelt relative to concentrations in all other hydrological pools (120 ± 16.9 µg/L). DOC concentrations were also relatively high (4.80 ± 3.74 mg-C/L), and likely associated with the flushing of DOC from soil surfaces during snowmelt runoff^26^.

#### Glacial inflows

At glacier termini, meltwaters were cold (1.04 ± 0.13 °C), circumneutral (pH = 7.33 ± 0.162) and with low suspended sediment concentrations (64.6 ± 24.7 mg/L; Table S3). Waters were fully oxygenated, suggesting little influence of anoxic subglacial waters, or at least partial open system conditions under the ice^27^. Downstream of the glaciers at the river deltas, waters were warmer (8.01 ± 0.42 °C), more alkaline (pH = 7.86 ± 0.08) and contained much higher suspended sediment concentrations (TSS = 562 ± 163 mg/L) before flowing into Lake Hazen.

**Figure 2:**
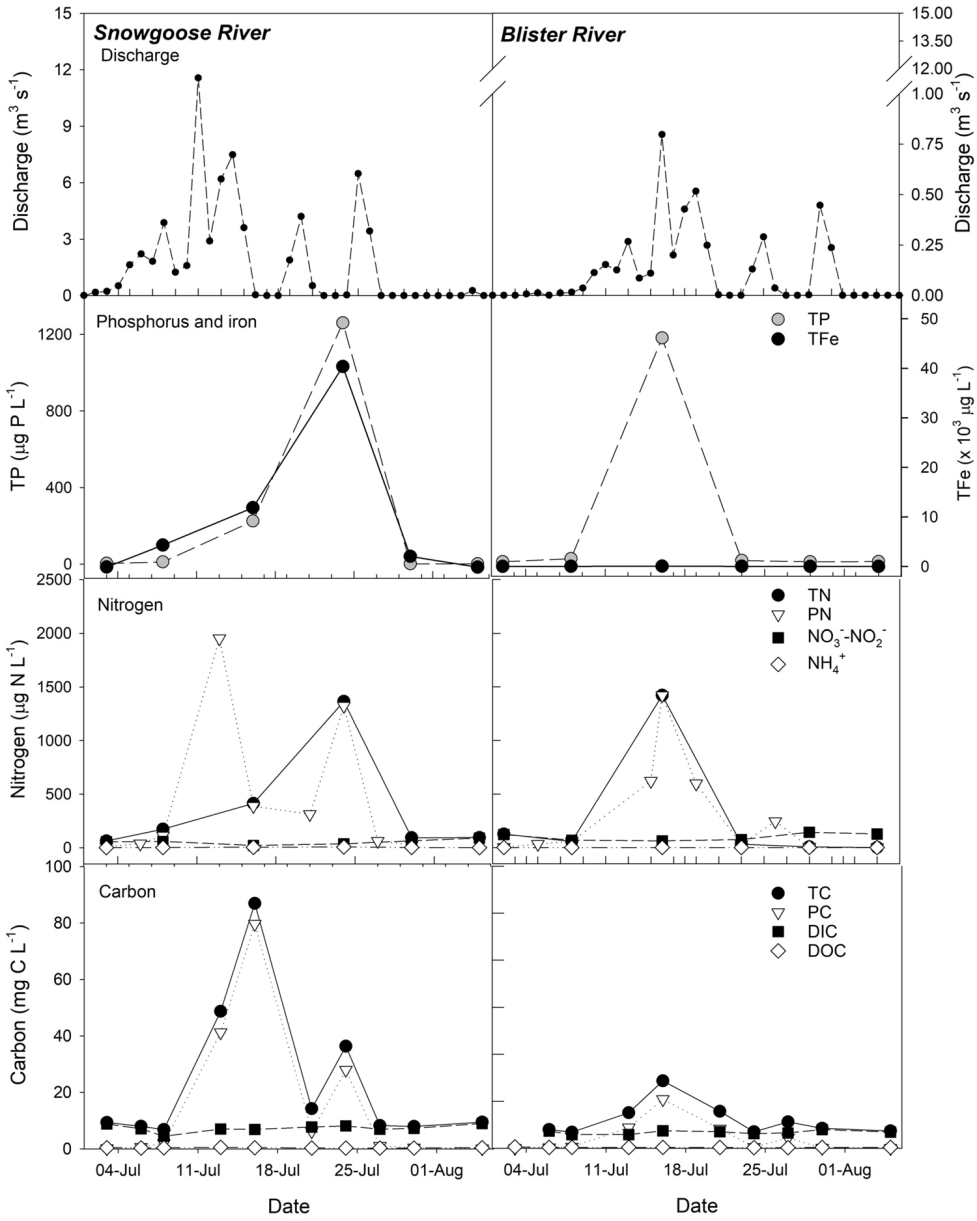
Time series of glacial river (Snowgoose and Blister) discharge (modelled) and chemistry measured at the inflow to Lake Hazen throughout summer 2016 (2-July to 4-August). Note that Blister River discharge was extrapolated from that of the Snowgoose River (see methods).

Nutrient concentrations, as well as SO_4_^2−^, dSiO_2_, and TFe, varied spatially within and between glacial rivers, and temporally throughout the melt season (Table S4). Concentrations of both dissolved and particulate constituents increased downstream of glaciers (Fig. S1), largely due to the erosion of highly reactive, comminuted sediments and subsequent chemical weathering^28^. With increasing discharge, dissolved constituent concentrations generally decreased with concomitant increases in particulate concentrations (Fig. 2), suggesting the adsorption of dissolved constituents to particles. Higher meltwater discharge entrains larger quantities of particulates as rivers erode channel edges and shifted positions across proglacial river valleys^29^.

DIN concentrations were higher in glacial river delta waters (75.6 ± 8.58 µg-N/L) than in other compartments of the summer watershed (p < 0.05, FDR-adjusted multiple comparisons) and dominated by NO_3_^−^-NO_2_^−^, except in the Gilman and Turnabout rivers, where NH_4_^+^ constituted more than half of the DIN pool (Table S4). TP concentrations in glacial rivers (661 ± 187 µg-P/L) were high and likely originated from marginal or proglacial zones^30^. Indeed, TP concentrations increased 2–3 orders of magnitude between the glacier termini and the lake (Fig. S1). TDP concentrations were below detection (<1.8 µg-P/L) in most glacial river samplings.

Early and late in the melt season, TC was dominated by DIC, but as glacial melt increased, PC became a proportionally greater fraction of TC (up to 92%; Fig. 2). In the Snowgoose River, for example, PC concentrations ranged between 0.04 mg-C/L early/late in the melt season to 79.7 mg-C/L at peak flow. DOC concentrations in glacial rivers were extremely low (0.3 ± 0.03 mg-C/L).

### In-lake structure and nutrient concentrations

#### Physical limnology

**Figure 3:**
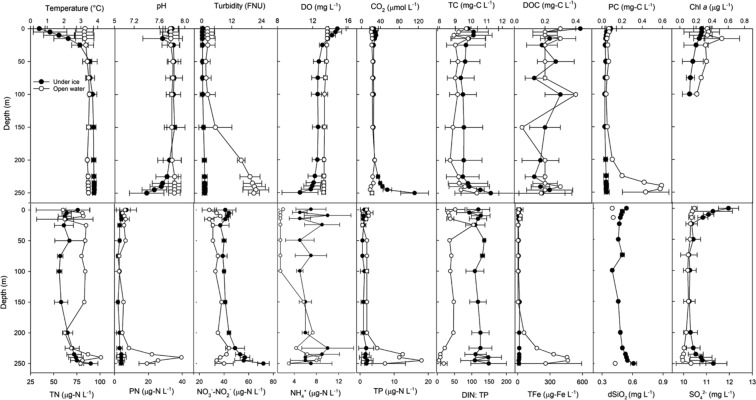
Seasonal physical and chemical water column profiles of Lake Hazen. Water column profiles averaged (±1 SD) by depth, depending on whether sampling was conducted under ice (May 2013, 2014 (2 profiles), 2017) or in open water (August 2015, 2016). O_2_, dissolved oxygen; DOC, dissolved organic carbon; PC, particulate carbon; Chl *a*, chlorophyll *a*; TN, total nitrogen; PN, particulate nitrogen; TP, total phosphorus; DIN:TP, dissolved inorganic nitrogen to phosphorus mass ratio; TFe, total iron; dSiO_2_, dissolved silica; SO_4_^2−^, sulfate.

Lake Hazen exhibited reverse temperature stratification under the ice, where temperatures near the surface were 0.52 ± 0.35 °C, but below 50 m, were constant at 3.72 ± 0.10 °C (Fig. 3). Thermal stratification disappeared following ice melt (3.37 ± 0.25 °C). Secchi depths before and after snowmelt were 27 m and 15 m, respectively, corresponding to light extinction coefficients of 0.063/m and 0.111/m (calculated from Secchi depth; Fig. S4). Turbidity in the water column in the spring was generally extremely low (Fig. 3), but increased marginally immediately following snowmelt in the spring.

In summer, turbidity varied spatially in Lake Hazen due to river inputs of glacial flour generated from erosion. Glacial flour severely impeded light penetration along the northwestern shoreline of the lake^31^. This effect was, however, limited to nearshore waters due to the density of inflowing glacial river waters; waters at the centre of the lake and along the southeastern shore away from glacial inputs were generally clear. With a Secchi depth of ~16 m, the light extinction coefficient at the centre of the lake was 0.106 /m, indistinguishable from that measured after snowmelt in the spring.

Glacial river discharge formed turbidity currents upon entering the lake, facilitating the transport of particulate-rich waters to depth and oxygenation of bottom waters (Fig. 3)^32^. Concentrations of PN and PC, proxies for TSS, increased 3-fold and 6-fold, respectively, between surface waters and the depths (200–250 m) of Lake Hazen, a subsidy from glacial inflows. Annual oxygenation of the bottom waters of Lake Hazen is a relatively recent phenomenon^10^, likely a consequence of enhanced glacial meltwater inputs to the lake. Indeed, the strength of the turbidity currents was directly dependent on the volume of glacial runoff in a given year. For example, glacial runoff in 2015 was 3.36 times greater than in 2016 (Table 1), mirrored by PC and TP concentrations at depth 2.20 and 3.83 times higher, respectively, in 2015 than in 2016.

#### Chemical limnology and potential nutrient limitation

74.0 ± 1.35% and 51.5 ± 2.52% of total nitrogen in the lake was DIN in spring and summer, respectively, mostly as NO_3_^−^-NO_2_^−^. Depth-integrated NO_3_-NO_2_^−^ concentrations in Lake Hazen were 49.7 ± 1.35 µg-N/L and 37.5 ± 1.74 µg-N/L in spring and summer, respectively (Fig. 3). These concentrations were lower than in more temperate lakes^33^, but up to 4.5-times higher than the mean NO_3_^−^-NO_2_^−^ concentration in other lakes in the Canadian Arctic Archipelago (CAA)^34^. The fact that Lake Hazen is glacier fed and so deep is unusual among the lakes previously studied in the CAA^34^, likely explaining the comparatively higher NO_3_^−^-NO_2_^−^ concentrations^11,12^. Although DIN concentrations were higher in the glacial rivers than in the lake waters, DIN in the summertime upper water column decreased. This likely reflected increased biological utilization at the surface, combined with the fact that glacial meltwaters rich in NO_3_^−^-NO_2_^−^ plummeted to the bottom of the lake with the turbidity currents.

**Figure 4:**
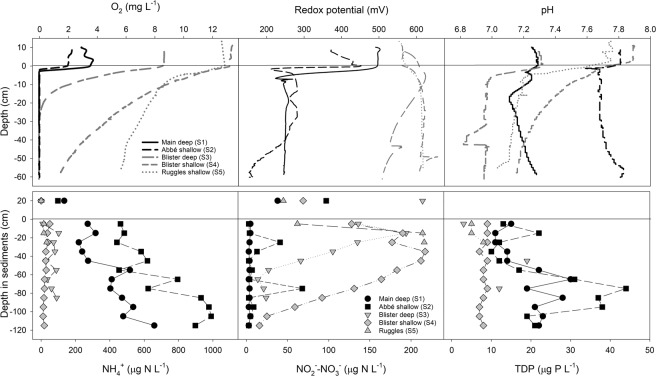
Sediment core microprofiles and porewater profiles of NH_4_^+^, NO_2_^−^NO_3_^−^ and total dissolved phosphorus (TDP) for 5 sites in Lake Hazen with variable proximity to glacial inflows. Cores nearer large glacial inflows are indicated in black. Concentrations in the overlying water were quantified on water samples collected from the top of the core tube before sectioning.

In spring under the ice, TP concentrations were below detection (<3 µg-P/L) throughout most of the water column, reaching a maximum concentration of only 6 µg-P/L near the sediment-water interface (Fig. 3). Although total dissolved phosphorus (TDP) was generally below detection (<1.8 µg-P/L) in the water column throughout the year, phosphorus can be mobilized into the water column from sediments via internal loading^35^. However, relatively consistent oxygenation of bottom waters likely limits the release of TDP, despite TDP concentrations in sediment core porewaters being much higher (up to 44 µg-P/L) than in the overlying water column (Fig. 4)^36^.

Mean DIN:TP mass ratios integrated throughout the springtime Lake Hazen water column were 55.7 ± 3.21 (Fig. 3), indicative of severe P-limitation^37^. In summer, the upper 200 m of the water column still exhibited strong P-limitation (DIN:TP = 21.3 ± 3.02); however, DIN:TP declined below 200 m to a minimum of 2.3, indicative of N and P co-limitation, or even possible N-limitation. These DIN:TP ratios at depth mirrored those in the glacial inflows (8.55 ± 4.84, median: 0.207; Table S3).

TC concentrations integrated throughout the water column were 9.40 ± 0.160 mg-C/L (summer) and 9.89 ± 0.150 mg-C/L (spring), ~98% of which was DIC (Fig. 2). DOC concentrations were extremely low throughout the water column: 0.228 ± 0.024 mg-C/L and 0.191 ± 0.016 mg-C/L in the summer and spring, respectively.

dSiO_2_ concentrations were low and constant throughout the water column throughout the year (Fig. 3). In contrast, while TFe was low or below detection (<7 µg/L) throughout the entire water column in spring, TFe increased 140-fold below 200 m in summer, reaching 495 µg/L. Summertime turbidity currents transport Fe to the depths from the watershed, where Fe is present in various mineral forms (e.g., sjögrenite, ankerite, pyrite, ferrinatrite).

### Nutrient concentrations in the Ruggles River outflow

The Ruggles River was cold (4.09 ± 0.291 °C), alkaline (pH = 7.96 ± 0.158) and fully oxygenated (105 ± 1.35% saturation) along its length. Waters were clear at the outflow of Lake Hazen (TSS = 1.40 ± 0.495 mg/L) but increased in turbidity (TSS up to 551 mg/L) with increasing distance from Lake Hazen due to permafrost slumping and erosion along the river banks (Fig. S2). This effect was particularly pronounced in early August when maximum permafrost thaw was occurring.

TN at the Lake Hazen outflow was primarily DIN (56.4 ± 16.7%), though concentrations were less than in the glacial rivers, and PN concentrations were very low, suggesting some organic nitrogen production within the lake (Table S3). TP concentrations at the lake outflow were 2.23 ± 0.959 µg/L, but increased up to 120-fold before entering Chandler Fjord. The river was P-limited at the lake (mass DIN:TP = 13.2 ± 6.38), but became N and P co-limited (2.88 ± 2.73) downstream due to erosional P inputs.

TC concentrations at the lake outflow were dominated by DIC (95.5 ± 1.04%), mirroring those in Lake Hazen, but PC became a more important component of TC downstream with slumping and erosion (up to 94.0% of TC). DOC was extremely low across the whole transect (0.30 ± 0.00 mg-C/L).

dSiO_2_ concentrations remained constant along the river. Mirroring other particulate parameters (Fig. S2), TFe concentrations in the Ruggles River increased downstream of Lake Hazen due to permafrost slumping and erosion (Table S3 and Fig. S3).

### Nutrient input-output budgets

Hydrological budgets for all chemical species, except NO_3_^−^-NO_2_^−^, NH_4_^+^, DIC and DOC, were generally net neutral, with annual Ruggles River exports approximately equaling the combined meltwater inputs. Glacial runoff accounted for most of all dissolved inputs to Lake Hazen (Table 1), but contributions varied inter-annually, such that glaciers accounted for a larger proportion of inputs (90.9 ± 2.53%) in the high melt year (2015), but only 78.0 ± 4.31% in the low melt year (2016).

Lake Hazen was a strong sink for NO_3_^−^-NO_2_^−^, and NH_4_^+^ (Table 1). Processes that could account for these sinks are biological uptake, sedimentation or the denitrification of NO_3_^−^ and subsequent evasion to N_2_O and/or N_2_^38^. Given that denitrification would be limited by the low temperatures and largely aerobic conditions in Lake Hazen^34,39^, the sedimentation of particle adsorbed-inorganic N and organic N is likely more important at the watershed scale. Anoxic conditions in bottom waters may lead to the release of stored N to overlying waters, but most of the water column is oxic, with the exception of the sediment-water interface at sites receiving large amounts of glacial inputs (Fig. 4). As oxygen is consumed by the aerobic degradation of organic matter within the sediments, the adsorptive capacity of the sediments is diminished (see below). For example, while concentrations of NH_4_^+^ in sediment porewaters were higher in the two cores characterized by high oxygen consumption rates (Fig. 4), NH_4_^+^ release from the sediments to overlying waters was likely limited by the consistent oxygenation of bottom waters^40^.

Lake Hazen was also a sink for DOC, but this was only significant in 2015 (Table 1), suggesting that the strength of the sink is strongly dependent on the volume of glacial inputs to the lake. Even though concentrations of DOC within all freshwater compartments of the watershed were low, the glacial rivers represent an important source of DOC to Lake Hazen due to the sheer volume of water entering the lake. Loss of DOC within the lake can occur by mineralization to CO_2_, photo-oxidation or adsorption to particles and subsequent deposition.

In contrast, Lake Hazen was a source of DIC to the Ruggles River. Within the lake, DIC could be generated through respiration, which produces CO_2_, and/or chemical weathering of mineral glacial flour, which produces HCO_3_^−^. While some respiration within Lake Hazen inevitably occurs, it is unlikely to be an important process in this ultra-oligotrophic lake. Indeed, CO_2_ concentrations throughout most of the water column (with the exception of bottom waters in the spring time; Table S2) were at or below saturation (Tables S2–3), in contrast to many other water bodies, where high respiration rates result in CO_2_ supersaturation^41^. Chemical weathering is therefore the likely source of the DIC increase within the lake. Glacial flour entering Lake Hazen is largely composed of carbonate minerals, a reflection of the dominant geology of the watershed^42^. As these finely comminuted carbonate sediments are mixed within the water column, they can undergo carbonate dissolution, which may or may not be coupled with sulphide oxidation^43^.

Given that the strength of the turbidity currents depends on the magnitude of glacial meltwater inputs in a given year, the lake was also likely a strong sink for particulate parameters (PC, PN, TFe, TP), even though we could not reliably calculate the glacial input component of these budgets. Although Lake Hazen is atypically large among Arctic lakes^44^, even small, more ephemeral proglacial lakes may be important modifiers of glacial meltwaters on northern landscapes^30,45^. We show here that proglacial lakes may act to retain and/or process nutrients before delivery and mobilization to ecosystems further downstream.

### In-lake biological processes

#### Algal biomass within Lake Hazen

Chl *a* concentrations were near detection throughout the year, regardless of season or ice cover on the lake. Below the lake ice, chl *a* concentrations more than doubled from 0.13 µg/L before snowmelt to 0.29 µg/L after snowmelt, likely reflecting an increase in chl *a* density per cell in response to decreasing light availability^46^ and/or an influx of nutrients in meltwaters. Chl *a* concentrations in the upper 25 m, though, were uniformly low (0.26 ± 0.01 µg/L; Fig. S5), as in other lakes found in the Canadian High Arctic^34^. Upper water column chl *a* concentrations in the summer were higher at the center of the lake (0.39 ± 0.048 µg/L) than along the shoreline (below detection to 0.13 µg/L), likely in response to reduced light availability along the shoreline near the turbid glacial inflows^47^. In 1958, primary production ranged from below detection to only 59 mg-C/m^2^/d even at the height of summer^48^. Although the location of the watershed within Quttinirpaaq National Park (est. 2000) precludes the repetition of these experiments using ^14^C, attempts to use non-invasive productivity techniques (e.g., diel O_2_, O_2_ stable isotopes) were unsuccessful, suggesting that productivity within the lake is still very low.

We identified 48 phytoplankton genera and over 60 species of phytoplankton in the Lake Hazen water column under the ice in the spring (Table S5), 33 of which were unique to that time of year. Chrysophytes, chlorophytes and, to a lesser extent, diatoms were the most abundant taxa in the upper water column (Fig. S5), although abundances of all taxa were extremely low. Cyanobacteria were also found at depths within the water column, but exhibited high spatial and inter-annual variability in abundances. Summer phytoplankton communities were less diverse (34 genera and 39 species, 15 of which were unique to the summer), but had higher biomass than those in the springtime (Fig. S5). Open water communities were dominated by cryptophytes, diatoms and chrysophytes, the latter of which are a hallmark of Arctic systems^49^ and successful competitors at low phosphorus concentrations^50^. Whereas chrysophytes were one of the most diverse taxonomic groups in Lake Hazen, the cryptophytes were represented by only three genera: *Rhodomonas -* often the dominant algae, *Cryptomonas*, and *Kateblepharis*. Both cryptophytes and chrysophytes can be mixotrophic and only photosynthesize under optimal conditions^39^. Indeed, the dominance of phytoflagellates in Lake Hazen in the summer is very similar to what is seen in large temperate lakes in winter^51^.

**Figure 5:**
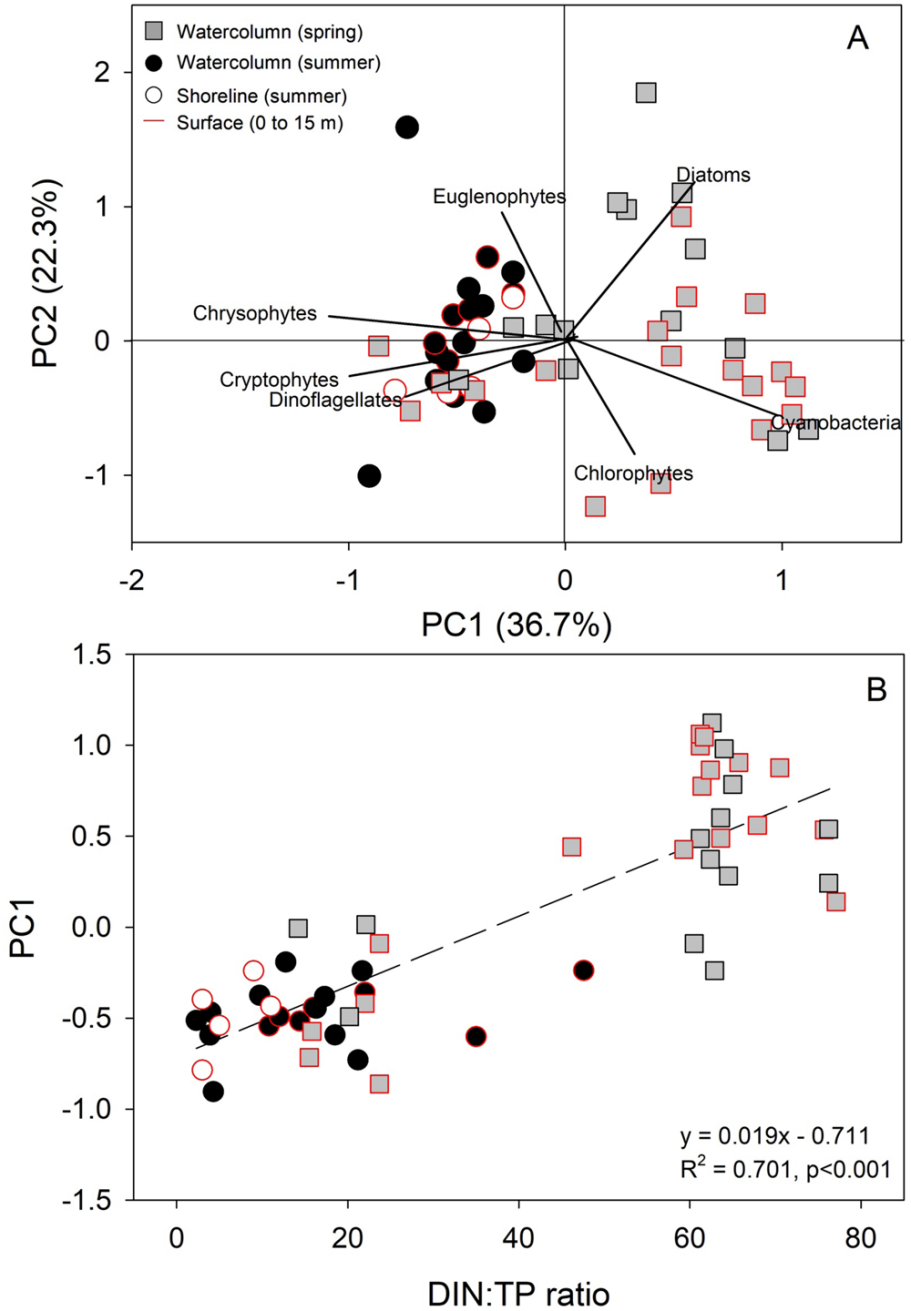
(**A**) Principal component bi-plot of phytoplankton community composition (as Hellinger-transformed cell densities) and (**B**) the first principal component (PC1) scores as a function of the DIN:TP mass ratio.

The overwhelming importance of DIN:TP in predicting phytoplankton community structure (both abundance and biomass) highlights the combined role of ice cover and glacial inflows on productivity in this ultra-oligotrophic lake (Figs 5 and S5). In fact, using cell densities as the response, the reduced RDA included only two variables: the DIN:TP mass ratio (RDA1 eigenvalue = 0.976) and CO_2_ saturation (0.120). In the spirit of parsimony, we therefore conducted a principal components analysis on the Hellinger-transformed cell densities as a metric of community composition, and regressed that against the DIN:TP ratio (Fig. 5; R^2^ = 0.701, p < 0.001). Whereas communities dominated by chrysophytes, cryptophytes and dinoflagellates, many of which are mixotrophic, were found at the lower DIN:TP ratios characteristic of open water conditions, higher abundances of diatoms, chlorophytes and cyanobacteria were found at the higher DIN:TP associated with the ice-covered water column.

Phytoplankton biomass was best predicted by DIN:TP and TP, water temperature and sulfate concentrations (Fig. S6). As these latter two variables play a comparatively small role on RDA2, they may reflect seasonality within the water column. Sulfate concentrations were higher under the ice in the spring (Fig. 3) and varied along the shoreline throughout the ablation season in response to glacial meltwater inputs. Similarly, temperatures were higher in the upper water column and along the shoreline during the summer relative to those under ice.

Even though it might seem counterintuitive that cyanobacteria would be more prevalent at higher DIN concentrations, only one genus of cyanobacteria possibly capable of nitrogen fixation was identified in the samples (*Aphanothece*). Whether the mixotrophic taxa (chrysophytes, cryptophytes, dinoflagellates) were autotrophic or heterotrophic at the time of sampling is unknown, but these taxa dominated along the shoreline where light penetration was significantly impeded by glacial flour and likely restricted primary production. Feeding strategy flexibility and motility in mixotrophic taxa likely provides a distinct competitive advantage in cold and unproductive Arctic freshwaters like Lake Hazen^52^.

While it has been suggested that increased glacial melt may enhance the productivity of downstream ecosystems^53,54^, we find little evidence of this within Lake Hazen, where phytoplankton cell densities, biomass and chl *a* concentrations were extremely low throughout the year. Indeed, because glacial meltwaters carry such large quantities of sediments and particulate-bound nutrients that (a) are not bioavailable, (b) impede light penetration along the shoreline of the lake, and (c) form turbidity currents which efficiently deliver these nutrients well out of the photic zone and to the bottom of the lake, enhanced glacial meltwater inputs may act to reinforce the ultra-oligotrophic status of the lake, rather than reduce it.

#### Zooplankton abundance and biomass

Our springtime zooplankton samples consisted entirely of juveniles that could not be identified to species. In the summer, ~98% of total zooplankton biomass was adult *Daphnia cf. galeata mendotae*. The balance consisted of nauplii, copepodid stages and adult cyclopoids (1.89% by weight; likely *Cyclops scutifer* as in ref.^55^) and the planktonic rotifer *Keratella hiemallis* (<0.1%). Copepods were, however, the most numerous zooplankton in Lake Hazen. Such low species richness is rare globally, but more common in alpine systems and the High Arctic, where extreme conditions preclude the development of many taxa^56,57^. At such low densities, zooplankton within the lake can only partially support the juvenile (<20% of diet) and small (<10% of diet) forms of the non-anadromous arctic char, which otherwise rely on chironomids and other terrestrial insects for the bulk of their diet^58^. Large char in the lake do not consume zooplankton, rather relying solely on cannibalism and chironomids as energy sources^58^.

#### Biogeochemical processes in sediments

OC accumulation rates in Lake Hazen sediments increased from ~8 to 14–71 g-OC/m^2^/yr in the past decade due to climate warming^10^, rates now similar to or greater than those found in temperate lakes^59^. Increased delivery of OC and other terrestrial materials to sediments have potentially important implications for both heterotrophic microbial activity and chemical oxidation processes there. Indeed, oxygen consumption rates varied depending on the proximity to and size of nearby glacial inflows delivering allochthonous materials to the lake. At the two sites receiving large inputs from the Snowgoose and Abbé rivers (Main deep, S1 and Abbé shallow, S2), dissolved O_2_ was depleted within 2–3 mm of the sediment-water interface, with a corresponding decline in redox potential, suggesting high levels of heterotrophic activity or chemical oxidation (Fig. 4). At the two sites presumably receiving less allochthonous material from the comparatively smaller Blister River (Blister deep and shallow, S3–4), dissolved O_2_ penetrated much deeper (25–>60 mm), with little change in redox. At the site receiving no direct glacial inputs (Ruggles, S5), dissolved O_2_ concentrations were still 6 mg/L at 60 mm depth. Depth-integrated O_2_ consumption rates were higher at those sites with higher bottom water O_2_ concentrations, reflecting the dependence of oxidation processes on O_2_ availability (Table S6).

Unsurprisingly, concentrations of NH_4_^+^ and TDP were highest at the sites receiving more glacial inputs, possibly reflecting higher rates of organic matter mineralization and consequent decreases in redox potential. Ammonification, as evidenced by high NH_4_^+^ concentrations, dominated in sediments with low O_2_ concentrations, whereas nitrification (higher NO_3_^−^-NO_2_^−^) dominated at the other sites. TDP was also highest in sediments with low dissolved O_2_, as phosphate (PO_4_^3−^) was likely being released from mineral sources and not trapped by Fe(III) precipitation under the prevailing reducing conditions.

In summer, when dense turbidity currents facilitated the oxygenation of bottom waters, dissolved O_2_ penetrated deeper into the sediments, with concomitant declines in NH_4_^+^ and TDP concentrations, and increases in NO_3_^−^-NO_2_^−^ (Fig. S7). Depth-integrated O_2_ consumption rates were an order of magnitude higher at both sites during the summer than in the spring, reflecting greater O_2_ availability at depth with lake mixing. For example, at S1, O_2_ consumption rates were 1.10 × 10^−3^ and 1.61 × 10^−4^ nmol/cm^2^/s in summer and spring, respectively, mirrored by rates of 2.56 × 10^−3^ and 3.63 × 10^−4^ nmol/cm^2^/s at S2. In the absence of stable reducing conditions during the summer months, nitrification prevailed over ammonification (lower NH_4_^+^, higher NO_3_^−^-NO_2_^−^), and the reduction and subsequent desorption of phosphorus from particles was diminished^60^.

### Land-to-ocean aquatic continuum in a glacierized watershed

**Figure 6:**
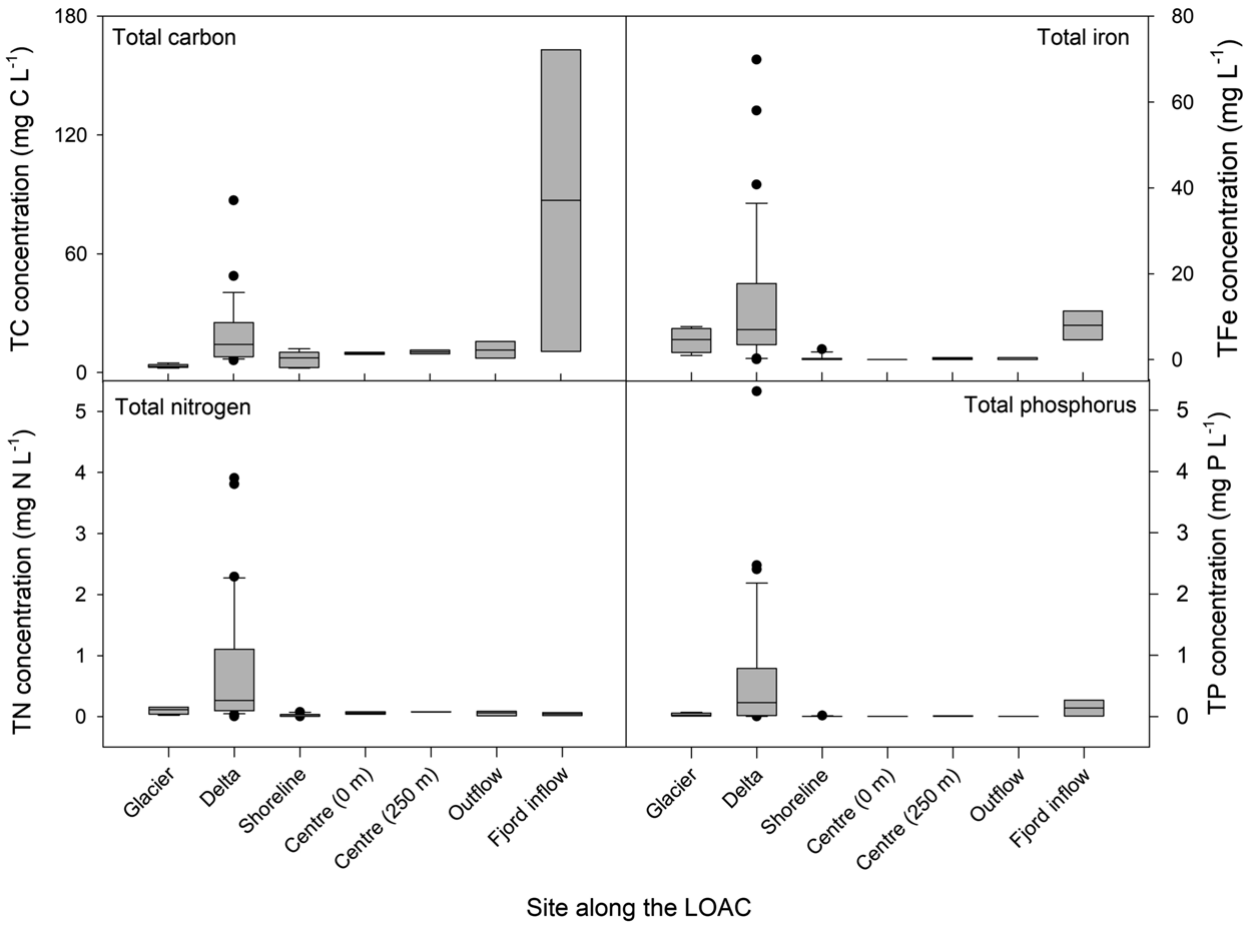
Nutrient concentrations along the land to ocean aquatic continuum (LOAC) in the Lake Hazen watershed. Sampling sites extended from immediately in front of the glacier termini to the glacial river deltas, along the shoreline of Lake Hazen, at the surface and bottom of the water column at the deepest (267 m) spot in the lake, along the Ruggles River outflow at Lake Hazen, and the Ruggles River inflow to Chandler Fjord along the northeastern coast of Ellesmere Island.

The glacierized LOAC observed in the Lake Hazen watershed and downstream is in stark contrast to boreal systems where carbon, nitrogen, and phosphorus concentrations decline according to first order decay^17^. TC, TP, TN, and TFe concentrations increased along our glacial rivers, with increasing availability of easily eroded fine materials downstream of glaciers and little retention within the proglacial landscape (Fig. 6). Whereas CO_2_ and CH_4_ emissions to the atmosphere tend to be an important carbon removal mechanism from non-glacierized freshwaters^41,61^, erosion and chemical weathering reactions in the glacier-fed freshwaters of the Lake Hazen watershed increase TC concentrations between glacial headwaters and the lake. Waters along the lake shoreline receiving glacial inputs acted as a physico-chemical intermediary between the rivers and central lake waters, where nutrient concentrations were much less variable. Due to turbidity currents transporting many of the glacial inputs directly to the bottom of the lake, the Lake Hazen outflow to the Ruggles River resembled that of central Lake Hazen surface waters. However, permafrost slumping and erosion downstream of the lake increased concentrations of all nutrients except TN, which lacks any important geological source, in the river.

**Figure 7:**
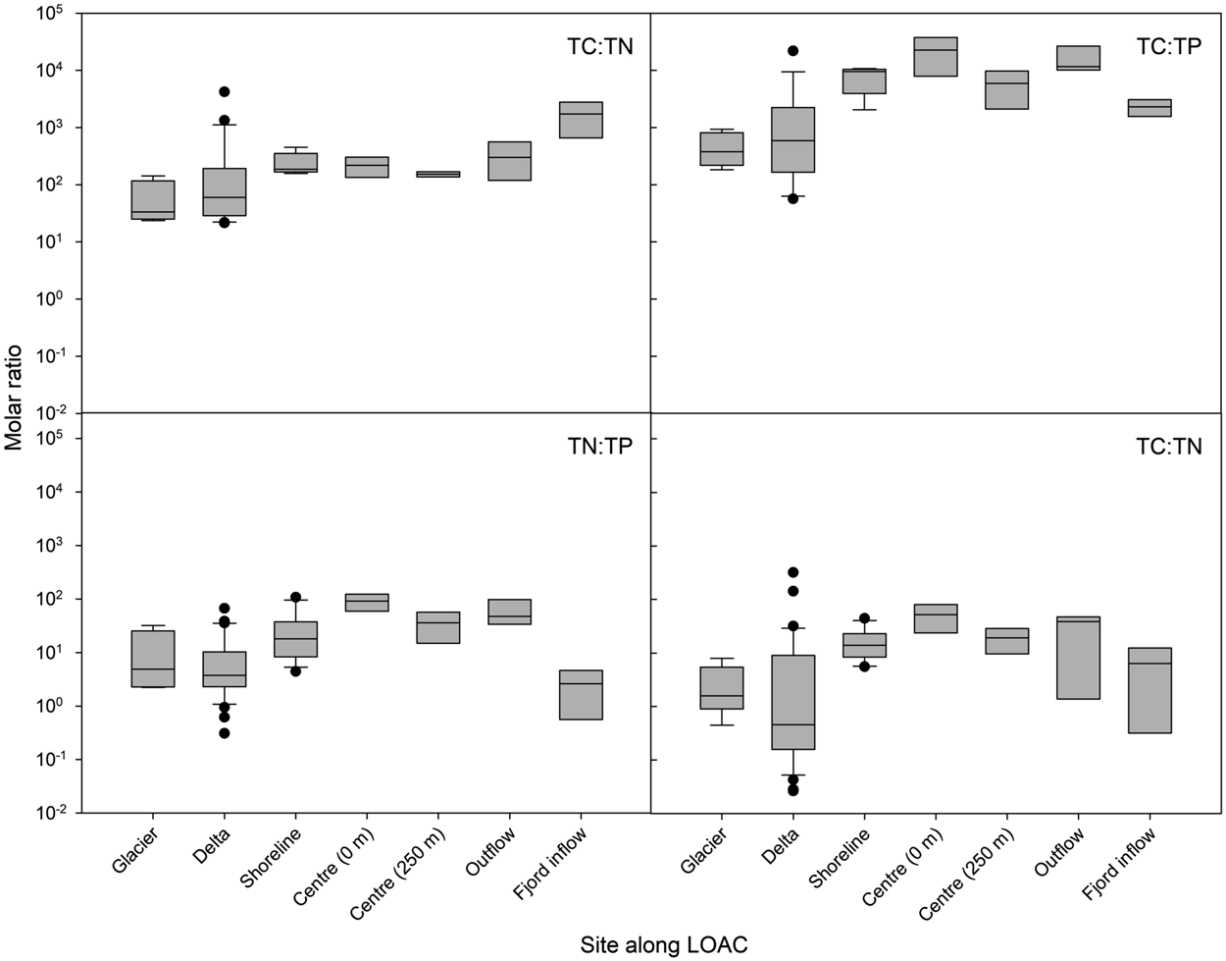
Molar ratios of key nutrients along the land to ocean aquatic continuum (LOAC) in the Lake Hazen watershed. Sampling sites extended from immediately in front of the glacier termini to the glacial river deltas, along the shoreline of Lake Hazen, at the surface and bottom of the water column at the deepest (267 m) spot in the lake, along the Ruggles River outflow at Lake Hazen, and the Ruggles River inflow to Chandler Fjord along the northeastern coast of Ellesmere Island. Note that the y-axis is on a log scale.

Stoichiometric ratios between carbon and key macronutrients (N, P) are useful tools to assess preferential nutrient loss or retention along the LOAC (Fig. 7)^62^. Unlike non-glacierized freshwaters, where biological processes exert a greater effect, nutrient stoichiometry across the Lake Hazen watershed was dominated by physical (hydrology, erosion, permafrost thaw and slumping) and chemical (weathering) processes, with important implications for downstream ecosystems. The combination of these processes led to the preferential mobilization of C, then P over N, the latter of which lacks any significant mineral source. Variability in glacial river delta stoichiometry reflected temporal fluctuations in meltwater volumes (Fig. 2) and spatial differences in geology across the watershed. TC:TP, TN:TP and DIN:TP in the Ruggles River declined before flowing into Chandler Fjord, highlighting mobilization of phosphorus by erosion and permafrost slumping to nearshore marine environments.

With the highest freshwater drainage to ocean basin area globally, the Arctic Ocean is especially sensitive to changes in freshwater quantity and quality from environmental change^63^. Although annual discharge from the Ruggles River (3.40 ± 1.85 km^3^/yr) is low compared to the 8 largest Arctic rivers (103–620 km^3^/yr)^64^, cumulative annual discharge from the Canadian Arctic Archipelago has been estimated to be 202–257 km^3^/yr^65,66^. Here we show that the quality of freshwaters transported downstream by the Ruggles River is strongly dependent on physical, chemical and biological processes occurring upstream across the watershed, as waters move from melting glaciers across the rapidly changing High Arctic landscape and through Lake Hazen. With continued warming, glacier melt and permafrost thaw in high latitude regions, our study suggests that these changes could have important implications for the health and productivity of Arctic aquatic ecosystems.

## Methods

### Site description

The Lake Hazen watershed, located within Quttinirpaaq National Park on northern Ellesmere Island, Nunavut, Canada (81–82°N, 70–72°W) is 7516 km^2^ in area, 40.9% of which is covered by outlet glaciers of the Northern Ellesmere Icefield. The watershed is underlain by a diverse geology, dominated by the largely sedimentary bedrock of the Cambrian Grant Land formation (quartzite, sandstone) along the northwestern shore and the Silurian Danish River formation along the southeastern Hazen Plateau (300–1000 m.a.s.l.), which lacks major ice or water features^42,67^. In summer, glacial meltwaters form braided rivers that flow between 4.6 and 42 km into Lake Hazen along its northwestern shore (Fig. 1). Lake Hazen itself sits at 157 m.a.s.l., and with a surface area of 544 km^2^ and maximum depth of 267 m, is the world’s largest High Arctic lake by volume (51.4 km^3^)^68^. Under current climatic conditions, ice cover on the lake begins to melt in early June, with complete ice loss, if it occurs, persisting from the end of July or early August to at least mid-September^10^. The Ruggles River (~29 km) flows year-round from the southeastern shore of Lake Hazen into Chandler Fjord on the northeastern coast of Ellesmere Island.

The watershed is a polar semi-desert receiving little precipitation throughout the year (~95 mm)^69^, ~65% of which falls as snow between September and May with the remainder as rain in the summer^70^. Snowmelt on the lake surface and adjacent landscape occurs over a 1–2-week period in early June. Meltwaters from the outlet glaciers of the Northern Ellesmere Icefield are by far the dominant hydrological input to the lake (Table 1), flowing between mid-June and the end of August. Small permafrost and ground-ice fed streams are found across the landscape but are hydrologically inconsequential at the watershed scale^10^ and generally only flow from late July for a few weeks and only in warmer years. Base flow in the Ruggles River is ~5 m^3^ s^−1^, increasing up to two orders of magnitude at the height of melt in mid-late July (Water Survey Canada station 10VK001, 1996–2012) following the onset of glacial river inputs to Lake Hazen^10^. The hydrograph of the outflow lags that of the combined inflows, though, such that Ruggles River discharge does not return to baseline until November or December, long after the glacial rivers cease flowing.

Sampling campaigns for this study were completed in spring (May/June) 2013, 2014, 2015 and 2017, and summers (July/August) 2015 and 2016 (Table S1).

### Hydrological input-output budgets

To understand the biogeochemical cycling of key nutrients across the Lake Hazen watershed, we constructed annual hydrological input-output budgets as follows:

1 $${{\rm{\Delta }}\mathrm{Storage}}_{{\rm{C}},{\rm{N}},{\rm{P}},{\rm{Si}},{\rm{Fe}}}={\rm{\Sigma }}\,{{\rm{Outputs}}}_{{\rm{C}},{\rm{N}},{\rm{P}},{\rm{Si}},{\rm{Fe}}}-{\rm{\Sigma }}\,{{\rm{Inputs}}}_{{\rm{C}},{\rm{N}},{\rm{P}},{\rm{Si}},{\rm{Fe}}}$$

∑ Inputs into Lake Hazen included snowmelt on the lake surface, snowmelt runoff from the landscape, and glacier melt from the Northern Ellesmere Icefield. Hydrological inputs from small permafrost and ground-ice fed streams were inconsequential at the watershed scale. ∑ Outputs from Lake Hazen is the export of nutrients via the Ruggles River. By convention, a negative ∆Storage indicates that Lake Hazen is a net sink (inputs exceed outputs) and a positive ∆Storage indicates that Lake Hazen is a net source (outputs exceed inputs) of the studied compounds. For each compartment within the watershed, we multiplied nutrient concentrations by water fluxes. This approach was applied to dissolved inorganic nitrogen species (DIN = NH_4_^+^ and NO_3_^−^-NO_2_^−^), SO_4_^2−^, dSiO_2_, dissolved organic carbon (DOC) and dissolved inorganic carbon (DIC). We could not estimate the budgets of particulate constituents (total carbon, TC; total phosphorus, TP; total nitrogen, TN; particulate nitrogen/carbon, PN/PC; total iron, TFe) due to challenges associated with quantifying the glacial flux component of these budgets for particulate parameters, described below. TC is calculated here as the sum of DIC, DOC and PC.

### Inputs to Lake Hazen

#### Snowmelt on the lake surface and from the adjacent landscape

Integrated snowpack samples were collected from the lake surface (20 sites total) and on the adjacent landscape (13 sites total) in May 2013, 2015 and 2017 using an acid-washed 4.3 cm diameter stainless steel corer. Snow samples were placed in large Ziploc^®^ freezer bags pretested for analyte contamination and kept frozen until processing and analysis.

Site-specific areal water volumes (AWV; L/m^2^) were determined from the weight of three snow cores and the diameter of the corer as follows:

2 $${\rm{AWV}}({\rm{L}}\,{{\rm{m}}}^{-{\rm{2}}})=\frac{{\rm{snow}}\,{\rm{weight}}\,(\mathrm{kg},\,{\rm{assuming}}\,{\rm{1}}\,{\rm{kg}}={\rm{1}}\,L)}{{\rm{\pi }}(\mathrm{corer}\,{\rm{radius}}\,{({\rm{m}}))}^{2}}$$

Snowmelt samples were collected from 3 streams in June 2017 only (n = 4 samplings). Temperature, pH, dissolved oxygen, and turbidity were measured continuously during sampling using an YSI EXO2 sonde. Bulk water samples were collected by dipping acid-washed 1-L Nalgene bottles and/or Platypus bags into the stream. Streams were sampled for the full suite of chemical constituents. Snowmelt runoff volumes were estimated by applying the mean ratio of SO_4_^2−^ concentrations in snowpacks (1.82 mg/L) and snowmelt (14.9 mg/L) in 2017, to the landscape AWV^71^. In assuming that all SO_4_^2−^ was conserved in the snowpack during melt, we calculate that 87.8% of snow from the landscape sublimated to the atmosphere, while 12.2% resulted in overland flow, as in other High Arctic watersheds^72^.

Snowpack depth and coverage were similar across all three years sampled, as well as for 2016, when snow was not collected (A. Ferguson, *personal communication*). For hydrological budget purposes, we applied 2015 AVW and snow chemistry to the 2015 budget and mean 2013, 2015, 2017 AVW and snow chemistry to the 2016 budget. Snow meltwater chemistry from 2017 was applied to snowmelt runoff volumes calculated from landscape AVW and the runoff ratio, as estimated above.

#### Glacial meltwater rivers

Water samples from the deltas of seven glacial inflow rivers (Snowgoose, Blister, Abbé, Gilman, Turnabout, Henrietta Nesmith, Very) were collected in the summers of 2015 and 2016 (Fig. 1). The Blister and Snowgoose rivers, which were within hiking distance of the base camp, were sampled weekly in both summers to assess seasonal variations in glacial river inputs to Lake Hazen (n = 5 in 2015 and n = 6 in 2016). The remaining glacial inflow rivers and the Ruggles River were accessed by helicopter once in 2015 (15-July) and twice in 2016 (11–13 July; 1–2-August). To assess biogeochemical changes along the length of the rivers, water samples were also collected at three sites between the glacier termini and Lake Hazen along the Blister, Snowgoose and Gilman rivers, and between Lake Hazen and Chandler Fjord along the Ruggles River. River waters were sampled like snowmelt streams, described above.

River hydrology:The Lake Hazen watershed was divided into glacier sub-catchments for all glacial rivers studied, using the 1:50,000 Canadian Digital Elevation Model (CDEM; Natural Resources Canada) in ArcGIS 10.5. Glacial river discharge for each sub-catchment was then modeled using a glacier surface mass balance approach^73^. This approximation of discharge was validated by comparing measured Ruggles River discharge (monitored by Water Survey Canada from 1996–2012) against cumulative modeled glacier runoff in the watershed (see ref.^10^). The Blister River sub-catchment was too small to delineate using the CDEM, so we applied the modeled runoff from the nearby Snowgoose Glacier (in kg m^−2^ d^−1^) to the Blister Ice Cap area from the Randolph Glacier Inventory (6 km^2^)^4^ as an estimate of Blister River discharge.

Chemical constituent fluxes:We generated log-linear models relating river discharge to chemical loads using the rloadest package in R^74,75^. Daily modeled water discharge for each of the rivers were combined with measured concentrations of the dissolved chemical species in each river (SO_4_^2−^, NH_4_^+^, NO_3_^−^-NO_2_^−^, dSiO_2_, DOC). LOADEST model 1 or 2 was selected for each parameter, depending on which iteration minimized bias, while maximizing explanatory power (Table S7). Bias across all models was less than 16%, and all models were statistically significant (R^2^ > 0.94, p < 0.001).

Only hydrological input-output budgets for dissolved constituents could be reliably calculated, which unfortunately precludes our ability to calculate budgets for bulk or particulate parameters (TP, TN, PN, TC, PC, TFe, TSS). Braided glacial rivers are highly unpredictable, with frequent channel reorganization and episodic bursts of meltwater. As such, although concentrations of particulate parameters were positively correlated with meltwater volumes (e.g., Fig. 2), the relationships were highly irregular and resulted in models with high bias and low predictive power. TDP concentrations were below detection in 73% of glacial rivers samplings, and TDP loads to the lake from glacial inputs could therefore not be reliably calculated.

### Hydrological outputs from Lake Hazen–the Ruggles River

The Lake Hazen outflow to the Ruggles River was accessed by helicopter at the same time as the glacial inflows during summers 2015 and 2016 and by snowmobile in springs 2015 and 2017. To quantify changes to Lake Hazen outputs along the length of the river, 2 additional sites downstream of Lake Hazen (mid-way down the river and just prior to the waters exiting to Chandler Fjord) were also sampled during the 2016 sampling campaign (Fig. S3).

Due to frequent water level sensor failures, Water Survey discontinued continuous gauging of the Ruggles River in 2012. Between 1996–2012, the volume of water exiting Lake Hazen via the gauged Ruggles River was, however, well approximated by the cumulative modeled glacial river inflows^10^. We therefore summed the modelled glacial inflows and the snow meltwater volumes to approximate Ruggles River discharge in the two years presented here. This approach assumes little evaporation along the length of the glacial rivers or from the surface of Lake Hazen, which is ice-covered most of the year.

The Ruggles River is not easily accessible, so we applied mean concentrations of all chemical parameters, combining samplings in springs 2015 and 2017 and summers 2015 and 2016, to the combined discharge to estimate annual Ruggles River exports from Lake Hazen.

### Lake sampling

#### Water column sampling

We sampled the water column of Lake Hazen at its deepest spot (~267 m) from the ice in May/June 2013, 2014, and 2017, and from a boat in August 2015 and 2016 to assess seasonal differences (with and without glacial inputs) in water column chemistry. Two water column profiles were completed in May/June 2014, to assess changes to upper water column chemistry before and after snowmelt. Continuous measurements of temperature, pH, turbidity and dissolved oxygen throughout the water column were recorded with a YSI EXO2 sonde. Depth-specific bulk water samples for full chemical analyses and phytoplankton community composition were then collected using an acid-washed 12-L Teflon-lined General Oceanics Niskin bottle. Each time, 15 discrete depths were sampled with a greater focus on upper and bottom waters (0 (or 2 under ice), 5, 10, 15, 25, 50, 75, 100, 150, 200, 225, 235, 240, 245, 250 m), except in 2016 when inclement conditions allowed for only three depths to be safely sampled (0, 15 and 250 m).

#### Shoreline sampling

Lake waters within 5–10 m of the northwestern shoreline of Lake Hazen were sampled weekly during summers 2015 (7-July to 2-August, n = 5) and 2016 (2-July to 8-August, n = 6) to quantify the nearshore influence of glacial inputs. To minimize sediment resuspension within the water column, samples were collected from areas along the lake shore with rocky beds upstream of the entry point to the lake.

### Biological community analyses

Samples for phytoplankton analysis were fixed with ~1 mL Lugol’s solution (in 50 mL) upon return to base camp and kept cool until analysis. Phytoplankton communities, biomass and cell densities were identified and enumerated following standard protocols by Plankton R Us (Winnipeg, MB) (e.g.^76^). Phytoplankton were identified to at least genus, and species when possible for historical record purposes (see the supplementary information), and then grouped according to major taxonomic group (cyanobacteria, chlorophyte, chrysophyte, diatom, cryptophyte, dinoflagellate) for the analyses here.

Relative influences of physicochemical variables on phytoplankton community composition were quantified using redundancy analysis (RDA)^77^, with the package *vegan*^78^. The eigenvalue of the first ordination axis in a preliminary correspondence analysis on the environmental variables was less than 0.6, supporting the use of linear methods and the RDA^77^. Environmental variables were standardized before running the saturated model and taxonomic cell density or biomass, proxies for growth or abundance, respectively, were Hellinger-transformed. The model was then run on the response covariance matrix. Variance inflation factors for environmental variables in the saturated model were greater than 10, indicating serial autocorrelation within the full model, and justifying our use of stepwise forward selection to identify the most parsimonious model using Akaike Information Criterion. Results presented herein are for the reduced models only.

Zooplankton were collected in spring 2014 and summer 2015 using a Dynamic Aqua 80 µm Wisconsin-style plankton net from the upper 75 m of the water column, and subsequently preserved with ethanol. In spring 2014, zooplankton were identified at Queen’s University, while in summer 2015, zooplankton were identified by Plankton R Us. Summer biomass estimates were obtained by measuring individual zooplankton and applying published length-weight regressions (e.g.^79–81^,).

The state of the arctic char (*Salvelinus alpinus*) population, the only fish species in Lake Hazen, has been described in detail elsewhere (e.g.^58,82–86^).

### Biogeochemical processes in Lake Hazen sediments

To assess the impacts of glacial meltwater inputs on heterotrophic and geochemical processes in lake sediments, sediment cores with intact sediment-water interfaces were collected in August 2016 and May/June 2017 using a UWITEC gravity corer (UWITEC, Mondsee, Austria) and 8.6 cm diameter polyvinyl chloride core tubes. Coring sites were chosen to encompass a variety of water depths and proximities to glacial river inflows (Table S8). Cores were collected at shallow (~50 m) and deep (~250 m) sites adjacent to the deltas of the Snowgoose-Abbé (S1, S2 in Fig. 1) and Blister (S3, S4) rivers, to represent high and low sediment inputs, respectively. We also sampled an additional shallow site near the Ruggles River outflow (S5) that received virtually no direct terrestrial runoff. Two cores were collected at each site.

Immediately upon return to base camp, 100-µm resolution microprofiles of O_2_, redox and pH were quantified on one of the cores using Unisense glass microelectrodes interfaced with the Unisense Field Microsensor Multimeter (Unisense, Aarhus, Denmark). Cores were maintained at ambient temperatures (~4 °C) throughout profiling. Microprofiles began either 1 or 2 cm above the sediment-water interface. Following microprofiling, the core was sectioned at 1 cm intervals to estimate porosity ($\phi )$ by weighing sections before and after freeze-drying, assuming all voids were water-filled prior to drying:

3 $$\phi ={{\rm{Volume}}}_{{\rm{voids}}}/{{\rm{Volume}}}_{{\rm{total}}}$$

Porewaters were extracted from the second core also upon return to the base camp. 1 cm core sections were placed in 50 ml centrifuge tubes, the headspace flushed with UHP N_2_ and centrifuged for 15 minutes at 4000 rpm (5018 g based on a 28 cm diameter rotor). The supernatant was then filtered through 0.45-µm cellulose-acetate (C/A) filters into 15 ml centrifuge tubes and immediately frozen for analysis of NO_3_^−^-NO_2_^−^, NH_4_^+^, and TDP (Table S9).

Sediment oxygen consumption rates were then estimated using the application PROFILE^87^. Sediment diffusion coefficients (D_s_) were calculated using:

4 $${{\rm{D}}}_{{\rm{s}}}={{\rm{D}}}_{{\rm{0}}}/(1+3\,\ast \,(1-{\phi }_{i}))$$

where D_0_ is the temperature-specific (3.76 °C at 250 m depth, n = 7 profiles) diffusion coefficient in water, and ${\phi }_{i}\,\,$is the site and depth interval-specific porosity for each depth interval. For sediment cores from sites S1–3, where oxygen was completely consumed over the profile, the bottom concentration (0 µmol /L) and bottom flux (0 nmol/cm^2^/s) were selected as boundary conditions. For the other sites (S4–5), the site-specific top and bottom concentrations were chosen as boundary conditions.

### Sample processing and chemical analyses

All samples were initially processed the day of collection in the analytical room of the Lake Hazen/Quttinirpaaq Field Laboratory, located at Parks Canada’s Lake Hazen Base Station. Upon return to base camp, water samples for DIN, TDP, SO_4_^2−^, dSiO_2_, DIC, and DOC were filtered through 0.45-µm C/A filters. Known volumes of water were filtered through muffled 0.7-µm GF/F filters for particulate carbon and nitrogen (PC and PN) analyses and pre-weighed 0.45-µm cellulose acetate filters for total suspended solids (TSS), analyses. Bulk water samples were collected for total phosphorus (TP) and total nitrogen (TN) analysis. For lake water samples, known volumes of water were also filtered through ethanol-washed 0.7-µm GF/F filters for chlorophyll *a* (chl *a*) concentrations. Samples were stored either frozen (chlorophyll *a*, NH_4_^+^, NO_3_^−^-NO_2_^−^) or in the dark at ~4 °C (all other samples) until transported south for analysis.

All chemical analyses were subsequently completed within ~2 months of collection at the Canadian Association of Laboratory Accreditation (CALA)-certified Biogeochemical Analytical Service Laboratory (BASL) at the University of Alberta (Edmonton, AB, Canada) following standard operating procedures (Table S1). For statistical purposes, analyte concentrations less than the method detection limit (<D.L.) were quantified as half of the D.L. (Table S1). Standard errors (SE) are presented throughout, unless otherwise stated.

## Supplementary information

Limnology of Lake Hazen Supplementary Information

Dataset 1

## Data Availability

Data will be made available by the authors upon request. Phytoplankton community datasets are available in the supporting information.
