# Supplementary material for: Contemporary limnology of the rapidly changing glacierized watershed of the world’s largest High Arctic lake
Source: Sci Rep. 2019 Mar 14;9:4447. doi: 10.1038/s41598-019-39918-4 (PMC6418217; doi:10.1038/s41598-019-39918-4)
Supplement: Supplementary file 1 — Limnology of Lake Hazen Supplementary Information [file 41598_2019_39918_MOESM1_ESM.pdf]

Supplementary Information

Contemporary limnology of the rapidly changing glacierized watershed of the world's largest High Arctic lake

St. Pierre, K.A.[1,*]; St. Louis, V.L. [1]; Lehnherr, I.[2]; Schiff, S.L.[3]; Muir, D.C.G. [4]; Poulain, A.J. [5]; Smol, J.P. [6]; Talbot, C.[4]; Ma, M.[1]; Findlay, D.L.[7]; Findlay, W.J. [7]; Arnott, S.E.[6]

[1] Department of Biological Sciences, University of Alberta, Edmonton AB Canada, T6G 2E9
[2] Department of Geography, University of Toronto at Mississauga, Mississauga ON Canada, L5L 1C6
[3] Department of Earth and Environmental Science, University of Waterloo, Waterloo ON Canada, N2L 3G1
[4] Canada Centre for Inland Waters, Environment and Climate Change Canada, Burlington ON Canada, L7S 1A1
[5] Department of Biology, University of Ottawa, Ottawa ON Canada, K1N 6N5
[6] Department of Biology, Queen's University, Kingston ON Canada, K7L 3N6
[7] Plankton R Us, Winnipeg MB Canada, R2N 1M1

Corresponding author: K.A. St. Pierre, kyra2@ualberta.ca

This PDF file includes:

Figs. S1 to S7
Tables S1 to S9

Other supplementary materials for this manuscript include the following:

Datasets S1. Phytoplankton community survey data

Supplementary Figures

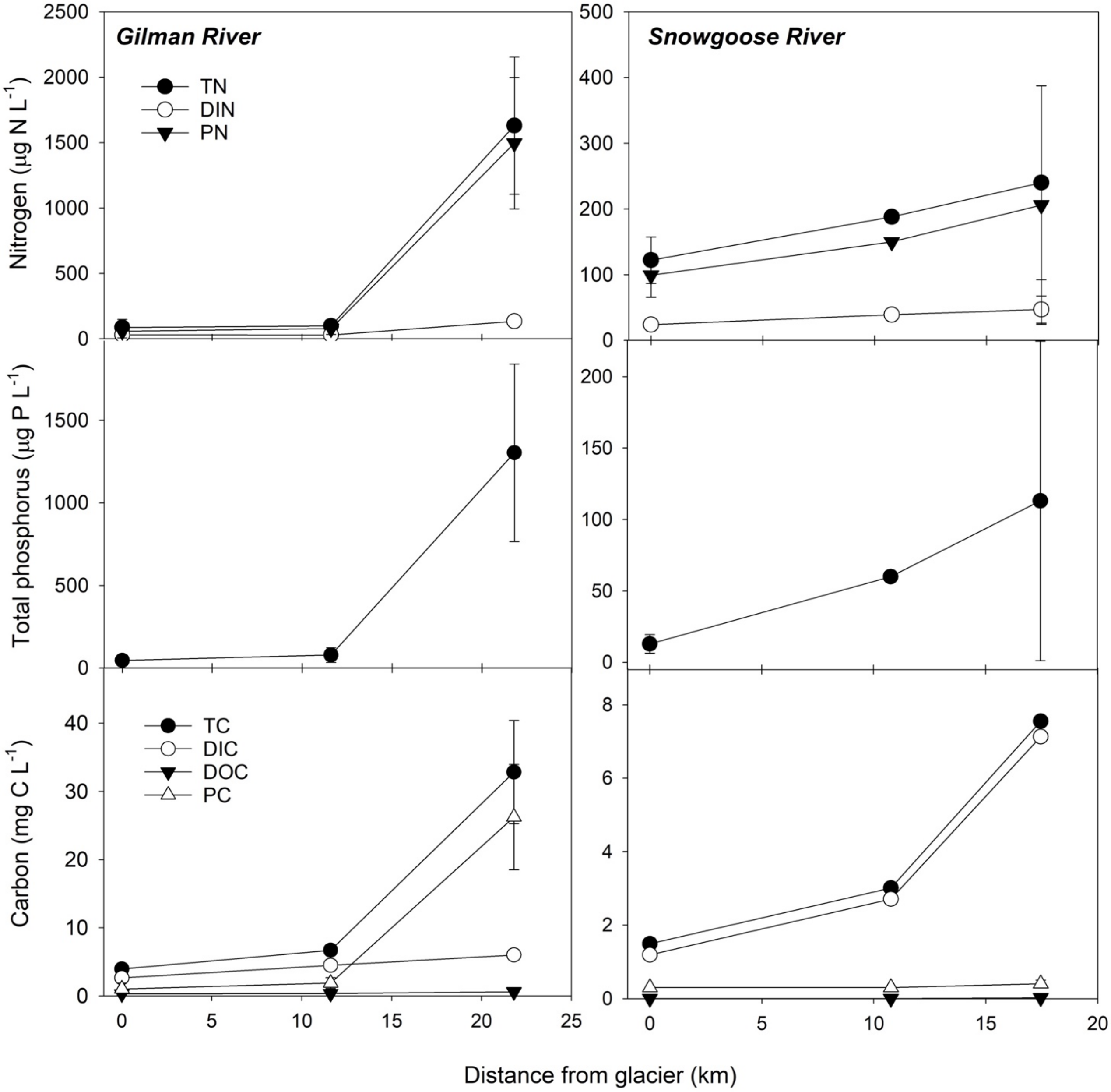

Fig. S1. Nitrogen, phosphorus and carbon transects along the Gilman (left panels) and Snowgoose (right panels) rivers in summer 2016 (means of 2 transects ± SE). TN, total nitrogen; DIN, dissolved inorganic nitrogen (sum of NH_4^+, NO_2^--NO_3^-); PN, particulate nitrogen; TC, total carbon; DIC, dissolved inorganic carbon; PC, particulate carbon; DOC, dissolved organic carbon. Transects conducted on 11-July and 1-August.

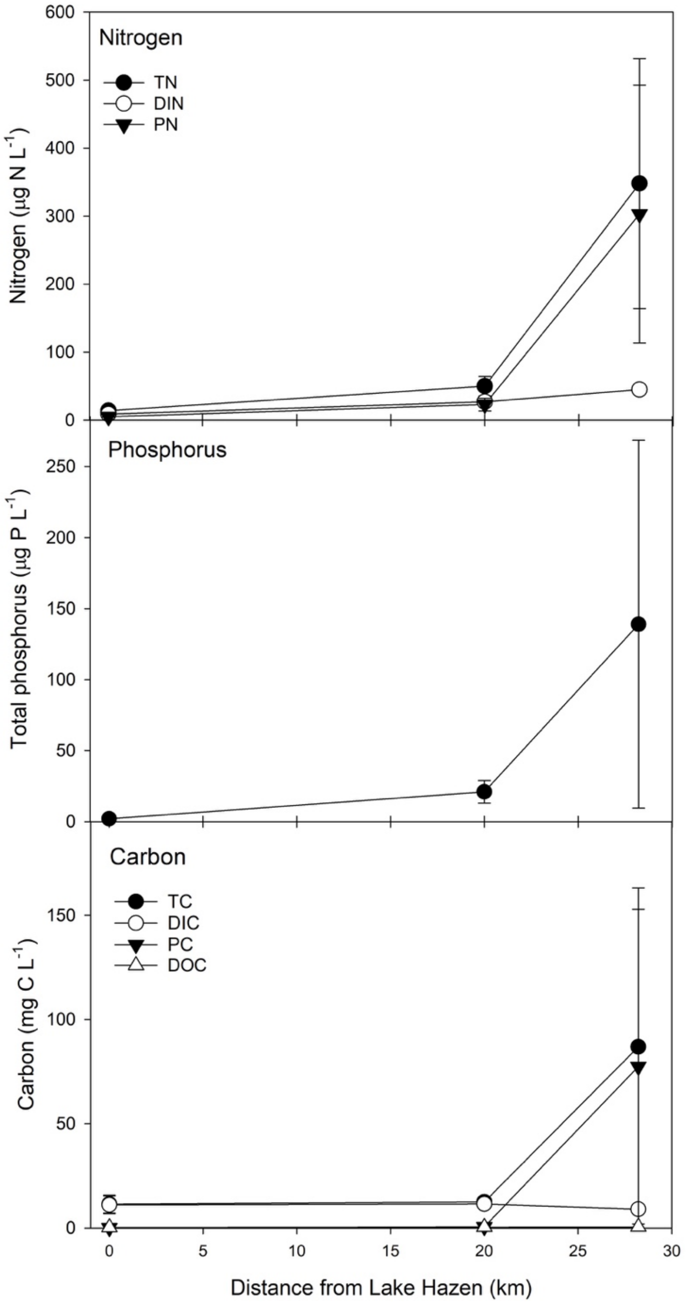

Fig. S2. Transects of nitrogen, phosphorus and carbon concentrations along the Ruggles River from Lake Hazen to Chandler Fjord in summer 2016 (means of 2 transects ± SE). TN, total nitrogen; DIN, dissolved inorganic nitrogen (sum of NH_4^+, NO_2^--NO_3^-); PN, particulate nitrogen; TC, total carbon; DIC, dissolved inorganic carbon; PC, particulate carbon; DOC, dissolved organic carbon. Transects sampled on 11-July and 2-August.

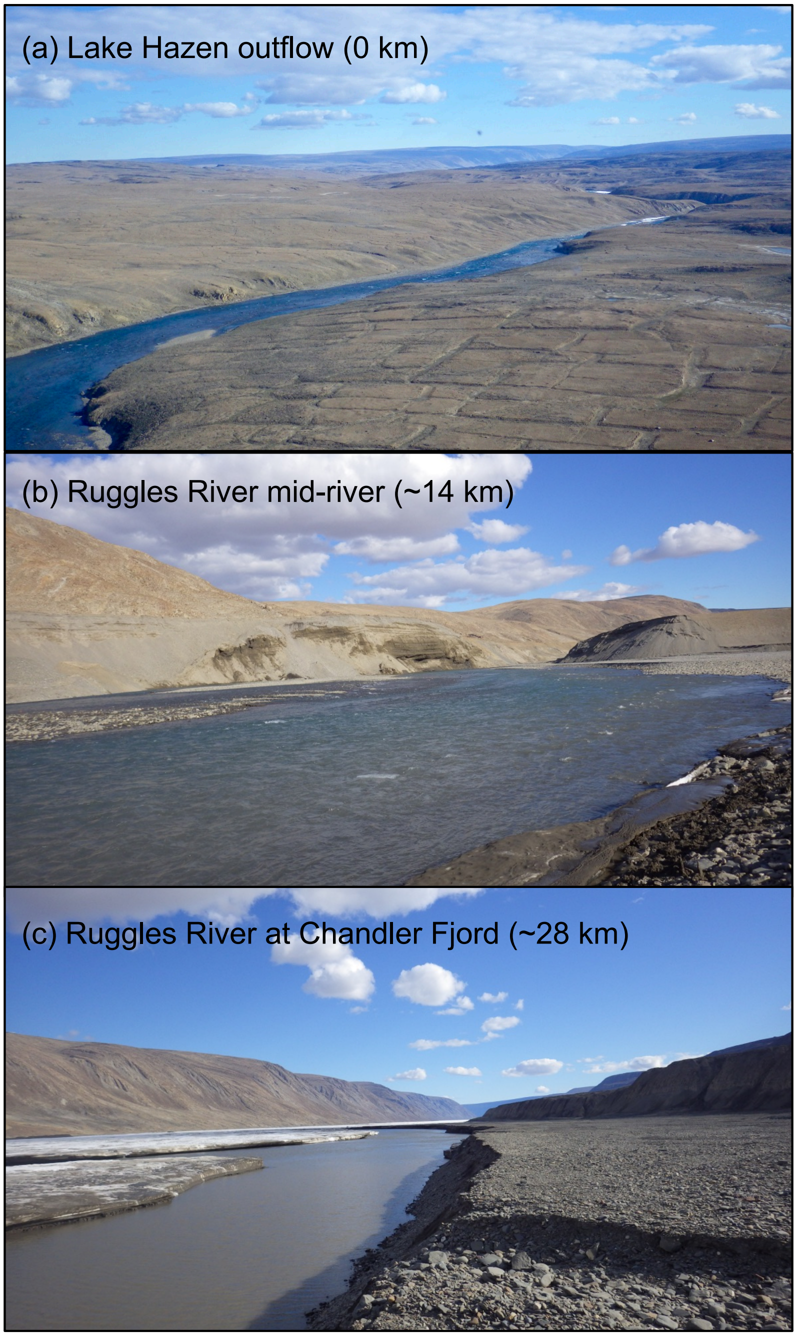

Fig. S3. Ruggles river transect sites with increasing distance from Lake Hazen (site location in brackets).

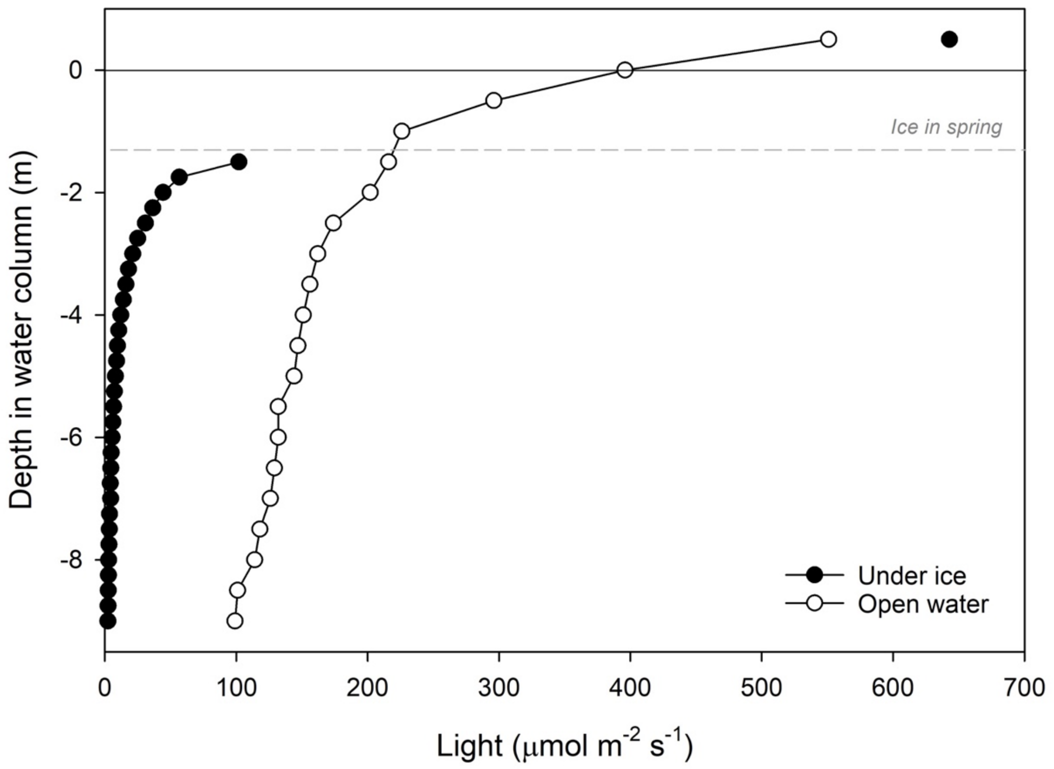

Fig. S4. Seasonal light profiles of the upper water column of Lake Hazen, measured using a Li-COR 1400 light meter.

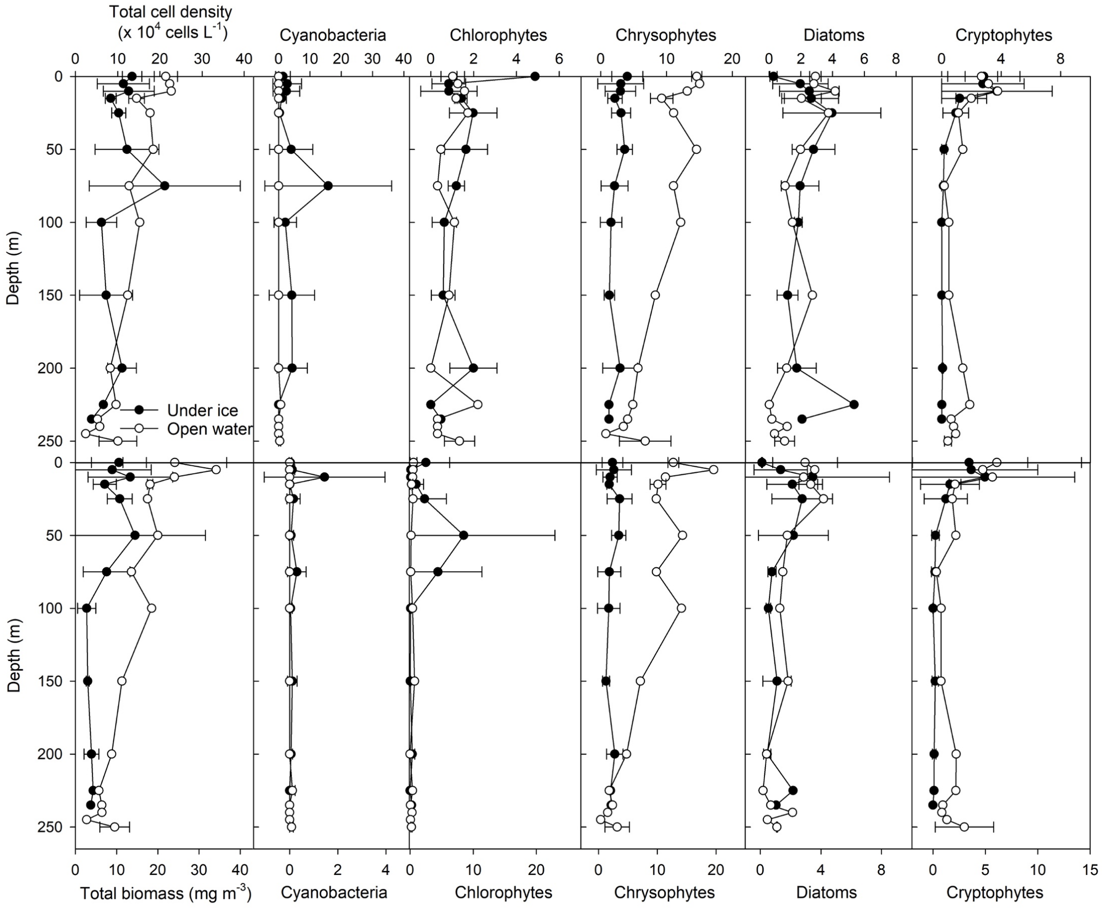

Fig. S5. Seasonal water column profiles of phytoplankton taxonomic group cell densities (x 10^4 cells L^{-1}, top panels) and biomass (mg m^{-3}, bottom panels) in Lake Hazen. Means (±1SD) by depth shown, over profiles conducted in May (under ice) 2014 (2 profiles), and in August (open water) 2015 and 2016.

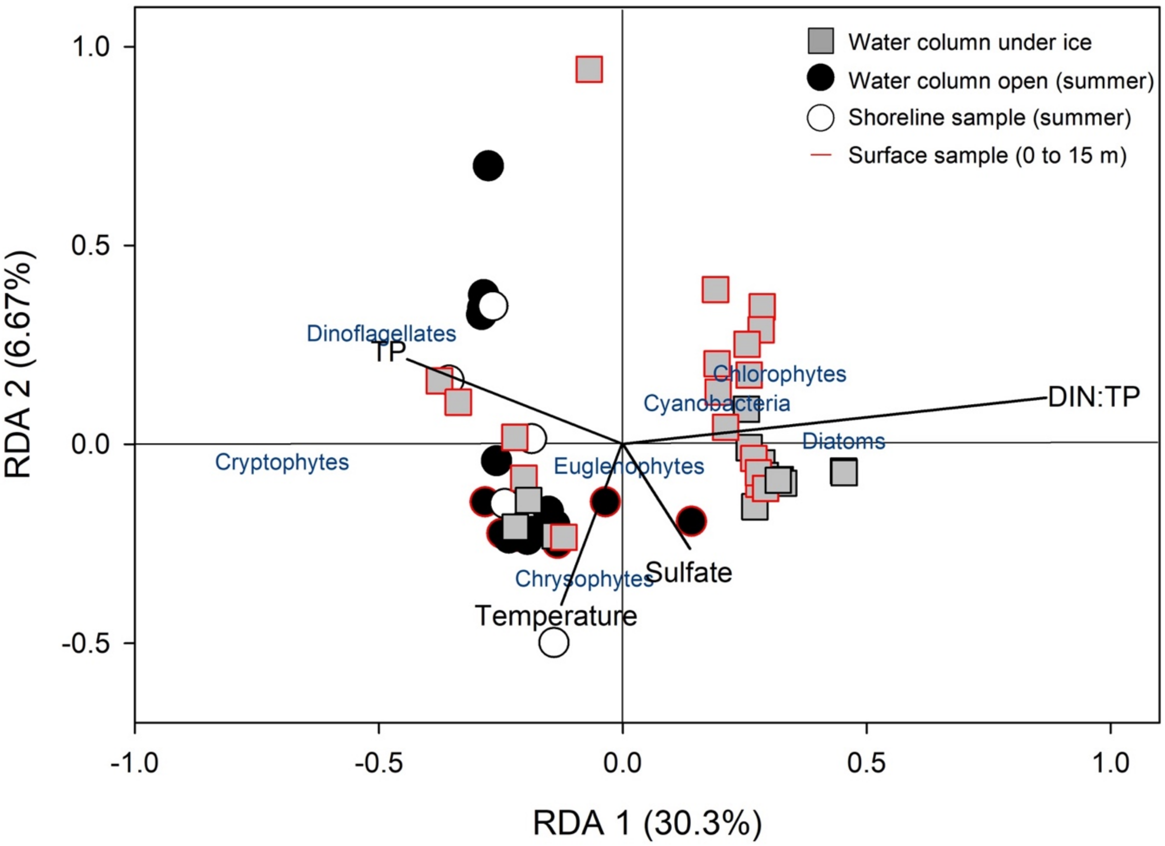

Fig. S6. Redundancy analysis of phytoplankton community composition by biomass.

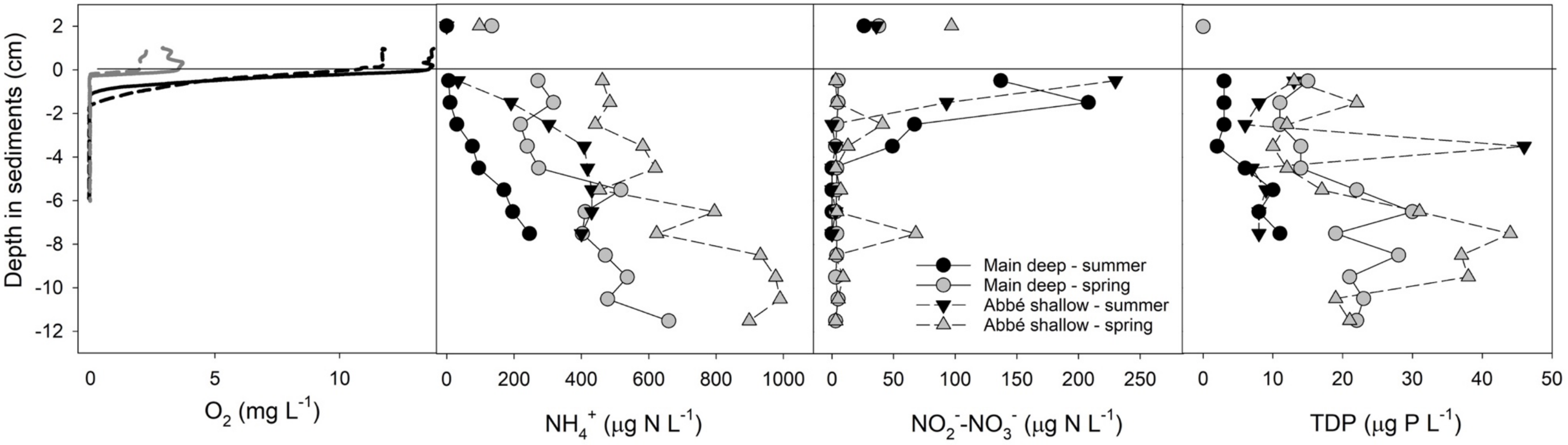

Fig. S7. Biogeochemical sediment core profiles from the glacially influenced main deep site (S1 in Figure 1) and Abbé shallow (S2, ~44 m) in August (black symbols) and the following May (grey symbols). Concentrations in the overlying water were quantified on water samples collected from the top of the core tube before sectioning.

Table S1. Sampling and analytical information for the Lake Hazen water column and glacial inflows.

				Snow	Snowmelt	Rivers		Lake Hazen water column				
				Spring	Spring	Summer			Spring		Summer	
**Parameter**	**Unit**	**Instrument**	**MDL**	**2013,15,17**	**2017**	**2015**	**2016**	**2013**	**2014**	**2017**	**2015**	**2016**
Temperature	°C	YSI EXO2 sonde	n/a		x	x	x	x	x	x	x	x
pH		YSI EXO2 sonde	n/a	x	x	x	x	x	x	x	x	x
Dissolved oxygen	mg L^{-1}	YSI EXO2 sonde	n/a		x	x	x	x	x	x	x	x
Dissolved carbon dioxide	µmol L^{-1}	Varian Chrompack CP-3800	n/a					x	x	x	x	x
Chlorophyll *a* (chl *a*)	mg L^{-1}	Varian Cary 50 Probe UV-Visible Spectrophotometer						x	x	x	x	x
Total suspended solids (TSS)	mg L^{-1}		0.05		x		x			x		x
Total dissolved species (TDS)	mg L^{-1}		0.04	x	x	x	x	x	x	x	x	x
Total nitrogen (TN)	µg L^{-1}	Lachat QuickChem QC8500	7	x	x	x	x	x	x	x	x	x
Particulate nitrogen (PN)	µg L^{-1}		n/a	x	x	x	x	x	x	x	x	x
Ammonium (NH_4^+)	µg L^{-1}	Lachat QuickChem QC8500	3	x	x	x	x	x	x	x	x	x
Nitrite + nitrate (NO_2^- + NO_3^-)	µg L^{-1}	Lachat QuickChem QC8500	2	x	x	x	x	x	x	x	x	x
Total phosphorus (TP)	µg L^{-1}	Lachat QuickChem QC8500	1.4	x	x	x	x	x	x	x	x	x
Total dissolved phosphorus (TDP)	µg L^{-1}	Lachat QuickChem QC8500	1.8	x	x	x	x	x	x	x	x	x
Total carbon (TC)												
Particulate carbon (PC)	mg L^{-1}		n/a	x	x	x	x	x	x	x	x	x
Dissolved organic carbon (DOC)	mg L^{-1}	Shimadzu TOC5000A Total Organic Carbon Analyzer	0.1	x	x	x	x	x	x	x	x	x
Dissolved inorganic carbon (DIC)[1]	mg L^{-1}		n/a	x			x	x	x		x	x
Sulfate (SO_4^{2-})	mg L^{-1}	Dionex DX-600 Ion Chromatograph	0.04	x	x	x	x	x	x	x	x	x
Dissolved silica ($dSiO_2$)	mg L^{-1}	Lachat QuickChem QC8500	0.02		x		x			x		x
Phytoplankton									x	x	x	x
Zooplankton									x		x	

[1] DIC in water samples were analyzed using the Varian Chrompack CP-3800; while DIC in snow samples were analyzed using a Shimadzu TOC-5000A Total Organic Carbon Analyzer.

Table S2. Spring physical and chemical limnology of hydrological compartments of the Lake Hazen watershed (mean of *n* ± SE).

	Watershed hydrological compartment				
			L.H. centre surface		
	Snow	Snowmelt	Before snowmelt	After snowmelt	L.H. bottom
n samplings [a]	*33*	*3*	*3*	*1*	*4*
**Physical**					
Temperature (°C)	-	1.09±0.500	0.189±0.050	0.933	3.709±0.085
pH	7.47±0.126	7.13±0.236	7.84±0.050	7.84	7.43±0.117
**Gaseous**					
O_2 (mg L^{-1})	-	14.3±0.185	15.4±0.152	8.80	10.0±0.580
CO_2 (µmol L^{-1}) [a]	-	-	33.4±1.63	9.59	112±14.7
**Bulk/particulate**					
TSS (mg L^{-1}) [a]	106±14.9	67.1±23.8	-	-	162±95.2
TN (mg-N L^{-1})	0.128±0.012	0.319±0.155	0.079±0.008	0.066	0.090±0.003
TP (mg-P L^{-1})	0.045±0.004	0.042±0.018	1.47±0.767	0.700	0.002±0.001
TFe (mg L^{-1})	0.222±0.081	4.12±0.011	<D.L.	<D.L.	<D.L.
PN (mg-N L^{-1})	0.059±0.014	0.198±0.013	0.004±0.001	0.016	0.004±0.001
PC (mg-C L^{-1})	2.10±0.194	1.97±0.495	0.060±0.011	0.170	0.048±0.013
**Dissolved (<0.45 µm)**					
NO_3^- (µg-N L^{-1})	41.6±2.63	84.3±23.8	45.6±0.865	27.7±0.333	72.0±1.68
NH_4^+ (µg-N L^{-1})	8.80±0.746	25.0±5.03	6.38±1.33	4.67±0.33	4.13±1.21
DIN (µg-N L^{-1})	50.4±3.12	109±18.8	51.3±2.67	32.3	75.2±3.17
DIN:TP (mass)	1.39±0.155	3.48±1.01	56.6±20.5	46.2	68.1±23.1
TDP (µg-P L^{-1}) *	2.52±0.308	-	<D.L.	<D.L.	<D.L.
DOC (mg-C L^{-1})	0.254±0.068	1.30±0.240	0.433±0.088	0.400	0.175±0.150
DIC (mg-C L^{-1}) [a]	1.94±0.115	-	10.2±0.723	6.79	11.0±2.04
SO_4^{2-} (mg L^{-1})	1.12±0.100	14.9±2.25	12.0±0.254	5.58	11.3±0.297
$dSiO_2$ (mg L^{-1})	0.085±0.006	0.24±0.020	-	-	-

[a] Snow means of 2013, 2015; before snowmelt mean of 2013, 2014, 2017; after snowmelt – 2014; bottom water mean of 2013, 2 x 2014, 2017.
* Calculated from concentration factor (see methods).

Table S3. Summer physical and chemical limnology of hydrological compartments of the Lake Hazen watershed (mean of *n* ± SE) averaged between summers 2015 and 2016.

	Watershed compartment						
	**Glacial headwaters**	**Glacial deltas**	**L.H. shoreline**	**L.H. centre surface**	**L.H. bottom**	**Ruggles outflow**	**Ruggles at fjord**
n samplings	*6*	*44*	*12*	*2*	*2*	*3*	*2*
**Physical**							
Temperature (°C)	1.04±0.130	8.01±0.42	5.00±0.537	3.23±0.371	3.46±0.04	3.75±0.286	4.00±0.127
pH	7.33±0.162	7.86±0.08	7.59±0.157	7.81±0.16	7.83±0.08	7.87±0.13	8.06±0.483
**Gaseous**							
O_2 (mg L^{-1})	14.5±0.399	12.0±0.147	12.4±0.043*	13.6±0.055	13.2±0.160	13.7±0.221	13.9±0.442
CO_2 (μmol L^{-1})	-	-	22.2±0.091*	22.5±2.06	22.1±2.73	-	-
**Bulk/particulate**							
TSS (mg L^{-1})	158±94.9	562±163	18.1±14.5	1.90	14.7	1.89±0.500	365±227
TN (mg-N L^{-1})	0.108±0.024	0.736±0.172	0.028±0.007	0.060±0.021	0.079±0.001	0.054±0.021	0.044±0.0245
TP (mg-P L^{-1})	0.046±0.020	0.661±0.187	0.003±0.001	0.002±0.001	0.007±0.004	0.002±0.001	0.139±0.130
TFe (mg L^{-1})	4.53±1.44	13.6±2.73	0.328±0.193	0.012±0.009	0.259±0.237	0.172±0.143	8.01±3.36
PN (mg-N L^{-1})	0.075±0.021	0.653±0.147	0.045±0.033	0.008	0.023	0.005±0.001	0.303±0.190
PC (mg-C L^{-1})	1.22±0.327	10.7±3.09	0.696±0.587	0.070±0.021	0.434±0.163	0.122±0.033	77.4±75.5
**Dissolved (<0.45 μm)**							
NO_3^- (μg-N L^{-1})	19.9±3.70	57.9±5.20	15.9±3.98	27.8±2.60	39.6±3.33	12.7±8.01	31.3±11.8
NH_4^+ (μg-N L^{-1})	5.97±0.958	17.7±6.39	2.99±0.685	1.92±0.274	5.70±1.28	4.83±1.69	13.3±5.8
DIN (μg-N L^{-1})	25.9±3.98	75.6±8.58	18.8±3.94	28.9±3.42	44.5±5.50	17.5±9.46	44.5±6.00
DIN:TP (mass)	1.29±0.601	8.55±4.84	6.43±1.11	22.9±12.1	8.55±4.20	13.2±6.38	2.88±2.73
TDP (μg-P L^{-1})	<D.L.	<D.L.	<D.L. to 3.0	9.5±2.5	11.5±5.5	<D.L.	<D.L.
DOC (mg-C L^{-1})	0.30±0	0.374±0.034	0.225±0.045	0.30±0.14	0.175±0.125	0.30±0.00	0.50±0.00
DIC (mg-C L^{-1})	1.73±0.373	7.00±0.261	5.51±1.41	9.34±2.31	9.77±5.23	10.3±2.61	8.99±0.749
SO_4^{2-} (mg L^{-1})	3.94±0.922	17.6±2.00	6.48±1.57	10.5±0.09	10.3±0.460	7.11±2.85	9.38±2.12
$dSiO_2$ (mg L^{-1})	0.080±0.003	0.290±0.029	0.248±0.074	0.400	0.430	0.260±0.080	0.305±0.085

Table S4. Physical and chemical limnology of the glacial river deltas in the Lake Hazen watershed (mean of *n* ± 1 SD) during summers 2015 and 2016.

	**Blister**	**Snowgoose**	**Abbé**	**Gilman**	**H. Nesmith**	**Turnabout**	**Very**
n samplings	*11*	*11*	*3*	*2*	*3*	*3*	*3*
**Physical**							
Length (km)	11.2	15.6	20.9	21.2	4.30	55.3	42.5
Watershed area (km^2)	-	222	390	992	1274	678	1035
Glacier area (km^2)	6	87	204	778	1041	259	269
Runoff ($km^3\ yr^{-1}$)		0.016 ± 0.014	0.038 ± 0.033	0.118 ± 0.105	0.183 ± 0.153	0.053 ± 0.041	0.122 ± 0.060
Temperature (°C)	8.90 ± 1.90	7.29 ± 1.53	5.96 ± 1.85	3.43 ± 0.281	6.27 ± 5.06	12.0 ± 0.943	10.3 ± 2.91
pH	7.80 ± 0.446	7.67 ± 0.634	7.94 ± 0.521	8.61 ± 0.157	8.16 ± 0.303	7.85 ± 0.181	8.18 ± 0.063
**Gaseous**							
O_2 ($mg\ L^{-1}$)	11.5 ± 0.61	12.0 ± 0.52	13.0 ± 0.19	14.0 ± 0.56	12.8 ± 1.76	11.2 ± 0.44	11.6 ± 1.08
**Bulk/particulate**							
TSS ($mg\ L^{-1}$) [b]	630±292	621±390	225±21.5	1064±154	64.8±10.2	547±367	275±36.9
TN ($mg\ N\ L^{-1}$)	0.401 ± 0.677	0.755 ± 1.03	1.55 ± 2.05	1.65 ± 0.716	0.201 ± 0.176	1.94 ± 1.68	0.554 ± 0.200
TP ($mg\ P\ L^{-1}$)	0.777 ± 1.64	0.408 ± 0.353	0.927 ± 1.34	1.30 ± 0.760	0.133 ± 0.171	0.408 ± 0.353	0.185 ± 0.169
TFe ($mg\ L^{-1}$)	8.64 ± 3.27	13.4 ± 4.35	25.8 ± 16.4	47.8 ± 22.1	5.72 ± 2.01	12.8 ± 3.40	5.74 ± 1.25
PN ($mg\ N\ L^{-1}$)	0.454 ± 0.677	0.526 ± 0.756	1.48 ± 2.03	1.49 ± 0.710	0.148 ± 0.157	1.73 ± 1.67	0.467 ± 0.186
PC ($mg\ C\ L^{-1}$)	4.40 ± 6.73	15.9 ± 26.5	5.66 ± 5.26	26.3 ± 10.9	5.85 ± 6.92	10.8 ± 7.37	10.0 ± 8.39
**Dissolved (<0.45 µm)**							
NO_3^--NO_2^- ($\mu g\ N\ L^{-1}$)	84.1 ± 31.7	46.6 ± 19.5	22.9 ± 5.89	44.0 ± 14.9	24.5 ± 2.83	71.1 ± 26.8	56.6 ± 19.8
NH_4^+ ($\mu g\ N\ L^{-1}$)	< 3.00	5.61 ± 3.76	6.50 ± 6.61	89.5 ± 17.7	7.25 ± 0.35	84.5 ± 95.6	23.5 ± 22.8
DIN ($\mu g\ N\ L^{-1}$)	85.9 ± 31.5	52.2 ± 17.2	29.4 ± 4.76	133 ± 32.5	31.8 ± 3.19	156 ± 122	80.1 ± 11.3
TDP ($\mu g\ N\ L^{-1}$) [a]	<D.L. to 6.00	< D.L. to 5.00	<D.L. to 8.00	<D.L.	<D.L.	<D.L. to 11.0	<D.L. to 3.00
DIN:TP (mass)	23.4 ± 46.9	2.28 ± 4.88	0.16 ± 0.16	0.11 ± 0.04	5.16 ± 6.61	1.36 ± 2.01	1.45 ± 1.87
DOC ($mg\ C\ L^{-1}$)	0.4 ± 0.1	0.4 ± 0.1	0.4 ± 0.1	0.6 ± 0.1	0.3 ± 0.0	1.1 ± 0.4	0.5 ± 0.1
DIC ($mg\ C\ L^{-1}$) [b]	6.79 ± 0.745	7.31 ± 1.26	5.31±0.577	6.002±0.383	4.96±0.119	8.34±0.129	9.72±0.526
SO_4^{2-} ($mg\ L^{-1}$)	23.7 ± 8.89	21.2 ± 16.2	11.8 ± 3.62	10.5 ± 0.714	11.2 ± 11.4	12.8 ± 3.89	14.0 ± 5.64
$dSiO_2$ ($mg\ L^{-1}$) [b]	0.43 ± 0.15	0.22 ± 0.09	0.14 ± 0.00	0.16 ± 0.02	0.11 ± 0.01	0.44 ± 0.01	0.30 ± 0.01

* TSS, total suspended solids; TN, total nitrogen; TP, total phosphorus; TFe, total iron; PN, particulate nitrogen; PC, particulate carbon. O_2, dissolved oxygen; CO_2, carbon dioxide; CH_4, methane; NO_3^-, nitrate; NH_4^+, ammonium; DIN, dissolved inorganic nitrogen; TDP, total dissolved phosphorus; DOC, dissolved organic carbon; DIC, dissolved inorganic carbon; SO_4^{2-}, sulfate; Cl^-, chloride; Na^+, sodium; K^+, potassium; Ca^{2+}, calcium; Mg^{2+}, magnesium; $dSiO_2$, dissolved silica.

[a] TDP presented as range due to fact that most measurements were below detection (<D.L.), where D.L. = 1.8 $\mu g\ P\ L^{-1}$.

[b] DIC, $dSiO_2$ and TSS concentrations are means from 2016 only.

Table S5. Seasonal phytoplankton genus and species diversity in Lake Hazen by taxonomic group.

	Total no.		Total under ice		Total open water		Unique under ice		Unique open water		Shared	
**Group**	**Genus**	**Species**	**Genus**	**Species**	**Genus**	**Species**	**Genus**	**Species**	**Genus**	**Species**	**Genus**	**Species**
Chlorophyte	10	11	8	9	8	8	2	3	2	2	6	6
Chrysophyte	16	21	12	17	10	11	6	7	4	4	6	10
Cryptophyte	3	7	3	4	3	5	0	2	0	2	3	3
Cyanobacteria	6	6	4	3	2	2	4	4	2	2	0	0
Diatom	17	23	17	20	6	8	11	13	0	2	6	8
Dinoflagellates	3	8	2	5	3	4	0	3	1	3	2	2
Euglenophyte	2	2	2	2	2	1	1	1	0	0	1	1
**Total**	57	78	48	60	34	39	24	33	9	15	24	30

Table S6. Overlying dissolved O_2 concentrations (1 cm overlying core ± SE), and maximum and depth-integrated oxygen consumption rates (in nmol cm^{-2} s^{-1}) for sediment cores collected throughout the Lake Hazen basin. See Table S3 for site information.

	SPRING			SUMMER		
**Site (ID)**	**Overlying O_2** µmol L^{-1}	**Integrated** x 10^{-4} nmol cm^{-2} s^{-1}	**Maximum** x 10^{-4} nmol cm^{-2} s^{-1}	**Overlying O_2** µmol L^{-1}	**Integrated** x 10^{-4} nmol cm^{-2} s^{-1}	**Maximum** x 10^{-4} nmol cm^{-2} s^{-1}
Main deep (S1)	104 ± 1.69	1.61	61.8	426 ± 0.59	11.0	3.81 x 10^{-3}
Abbé shallow (S2)	64.2 ± 0.76	3.63	26.9	362 ± 2.06	25.6	2.67 x 10^{-3}
Blister deep (S3)	270 ± 0.17	10.8	22.7	-	-	-
Blister shallow (S4)	414 ± 0.41	5.19	5.19	-	-	-
Ruggles River (S5)	398 ± 0.55	6.85	11.6	-	-	-

Table S7. LOADEST log-linear models for glacial inflow chemical fluxes, where [C] is in mg L^{-1} and Q is discharge in m^3 s^{-1}. All constituents are dissolved (< 0.45 μm).

Analyte	Model	R^2	p	Bias (%)
NH_4^+	$\ln[NH_4^+] = 0.004\ln Q^2 + 1.17\ln Q - 2.28$	0.937	<0.0001	-13.9
NO_3^--NO_2^-	$\ln[NO_3^- - NO_2^-] = 0.915\ln Q + 0.239$	0.979	<0.0001	-3.14
$SO_4^{2-}{}_{(aq)}$	$\ln[SO_4^{2-}] = 0.018\ln Q^2 + 0.903\ln Q + 5.92$	0.978	< 0.0001	-15.9
$dSiO_{2(aq)}$	$\ln[SiO_2] = 0.007\ln Q^2 + 0.905\ln Q + 1.06$	0.977	<0.0001	-0.78
DIC	$\ln[DIC] = 0.991\ln Q + 5.07$	0.996	<0.0001	15.5
DOC	$\ln[DOC] = 1.001\ln Q + 2.29$	0.973	<0.001	-1.83

Table S8. Sediment core sampling site information.

Site (ID)	Coordinates	Depth (m)	Large glacial influence (Y/N)	Years sampled<br>Summer 2016	 Spring 2017
Main deep (S1)	81.825°N, 70.715°W	255-267	Y	x	x
Abbé shallow (S2)	81.842°N, 70.852°W	26-44	Y	x	x
Blister deep (S3)	81.792°N, 71.469°W	251	N	-	x
Blister shallow (S4)	81.805°N, 71.527°W	53	N	-	x
Ruggles River (S5)	81.803°N, 70.504°W	60	N	-	x

Table S9. Summary of parameters measured on sediment cores collected from Lake Hazen.

Parameter	Instrument	Resolution (mm)	M.D.L.	Unit	Summer 2016	Spring 2017
Porosity	n/a	10	n/a		x	x
Dissolved oxygen (DO)	Unisense FMM	0.1		mg L^{-1}	x	x
pH	Unisense FMM	0.1			x	x
Redox potential (RD)	Unisense FMM	0.1		mV	x	x
Hydrogen sulfide (H_2S)	Unisense FMM	0.1		µmol L^{-1}	x	
Ammonium (NH_4^+)	Lachat QuickChem QC8500	10	3	µg L^{-1}	x	x
Nitrate + nitrite (NO_3^--NO_2^-)	Lachat QuickChem QC8500	10	2	µg L^{-1}	x	x
Total dissolved phosphorus (TDP)	Lachat QuickChem QC8500	10	1.8	µg L^{-1}	x	x
Sulfate (SO_4^{2-})	Dionex DX-600 Ion Chromatograph	10	0.04	mg L^{-1}	x	x

Additional data table (separate file)

Phytoplankton community composition data are appended in a separate file.
